# Plasmonic nanomaterials with responsive polymer hydrogels for sensing and actuation

**DOI:** 10.1039/d1cs01083b

**Published:** 2022-04-26

**Authors:** Fiona Diehl, Simone Hageneder, Stefan Fossati, Simone K. Auer, Jakub Dostalek, Ulrich Jonas

**Affiliations:** Macromolecular Chemistry, Department of Chemistry and Biology, University of Siegen Adolf Reichwein-Straße 2 57074 Siegen Germany jonas@chemie.uni-siegen.de; Biosensor Technologies, AIT-Austrian Institute of Technology GmbH Konrad-Lorenz-Straße 24 3430 Tulln an der Donau Austria jakub.dostalek@ait.ac.at; CEST Competence Center for Electrochemical Surface Technologies 3430 Tulln an der Donau Austria; FZU-Institute of Physics, Czech Academy of Sciences Na Slovance 2 Prague 182 21 Czech Republic

## Abstract

Plasmonic nanomaterials have become an integral part of numerous technologies, where they provide important functionalities spanning from extraction and harvesting of light in thin film optical devices to probing of molecular species and their interactions on biochip surfaces. More recently, we witness increasing research efforts devoted to a new class of plasmonic nanomaterials that allow for on-demand tuning of their properties by combining metallic nanostructures and responsive hydrogels. This review addresses this recently emerged vibrant field, which holds potential to expand the spectrum of possible applications and deliver functions that cannot be achieved by separate research in each of the respective fields. It aims at providing an overview of key principles, design rules, and current implementations of both responsive hydrogels and metallic nanostructures. We discuss important aspects that capitalize on the combination of responsive polymer networks with plasmonic nanostructures to perform rapid mechanical actuation and actively controlled nanoscale confinement of light associated with resonant amplification of its intensity. The latest advances towards the implementation of such responsive plasmonic nanomaterials are presented, particularly covering the field of plasmonic biosensing that utilizes refractometric measurements as well as plasmon-enhanced optical spectroscopy readout, optically driven miniature soft actuators, and light-fueled micromachines operating in an environment resembling biological systems.

## Abstract 2

The combination of plasmonic nanomaterials with responsive polymer hydrogels yields novel materials and devices with exceptional synergetic properties, which are reviewed in the context of applications, such as sensing and actuation.

## Graphical abstract

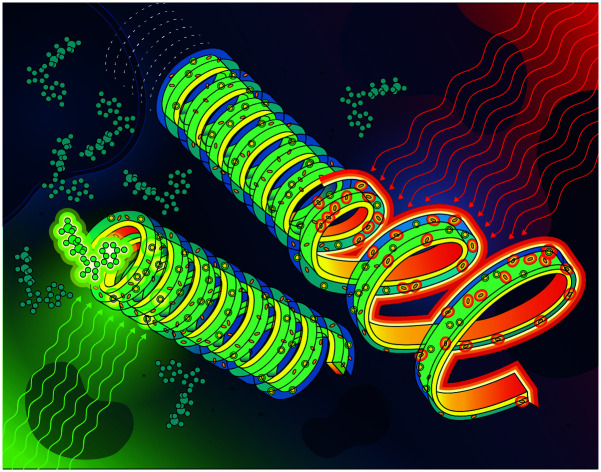

## 1. Introduction

Metallic nanoparticles and nanostructured thin metallic films exhibit unique optical properties associated with collective oscillations of electron density coupled with the respective electromagnetic field. These oscillations are referred to as surface plasmons, and their resonant optical excitation allows for tight confinement of light energy, strong increase of its intensity, and enhancement of local density of optical states.^1^ The progress in understanding of the design rules and gradual advancing of fabrication precision of plasmonic nanostructures paved the way towards a plethora of applications of surface plasmon resonance that range from efficient amplification of weak optical spectroscopy signal,^2,3^ analytical biosensor technologies for detection of minute amounts of chemical and biological species,^4–6^ light manipulation in highly miniaturized devices and optical integrated circuits,^7,8^ to systems serving for trapping of light in photovoltaic technologies.^9^ Metallic nanostructures can be prepared by chemical synthesis in liquid media and subsequently assembled into more complex architectures,^10^ or they can be directly fabricated by various lithography-based techniques on solid substrates.^11,12^ However, these metallic structures are typically static, meaning their optical characteristics are determined by the chosen geometry and typically cannot be reconfigured after the preparation step.

To further extend the spectrum of functionalities plasmonic materials can provide, on-demand tunable plasmonic architectures attracted a great deal of attention in the research community.^13^ For instance, inorganic materials with rapidly actuated refractive index have been proposed for fast plasmonic modulators.^14^ Moreover, we witnessed additional approaches based on decorating the metallic nanostructures with photoswitchable organic molecules such as azobenzene derivatives.^15,16^ A different approach that is particularly attractive in the bioanalytical field is based on merging metallic nanostructures with responsive hydrogels^17^ in order to harness their stimulus-sensitive characteristics and benefit from their biocompatible properties. Such responsive hydrogels are composed of water-swollen networks with interconnected responsive polymer chains, and their physical properties can be toggled between different states by applying an external stimulus.^18^ They can be prepared by numerous versatile synthetic routes^19^ and constitute an important class of soft matter with switchable features that already routinely serve in the prominent fields of biomedical devices, drug delivery systems, and tissue engineering.^20^

**Fig. 1:**
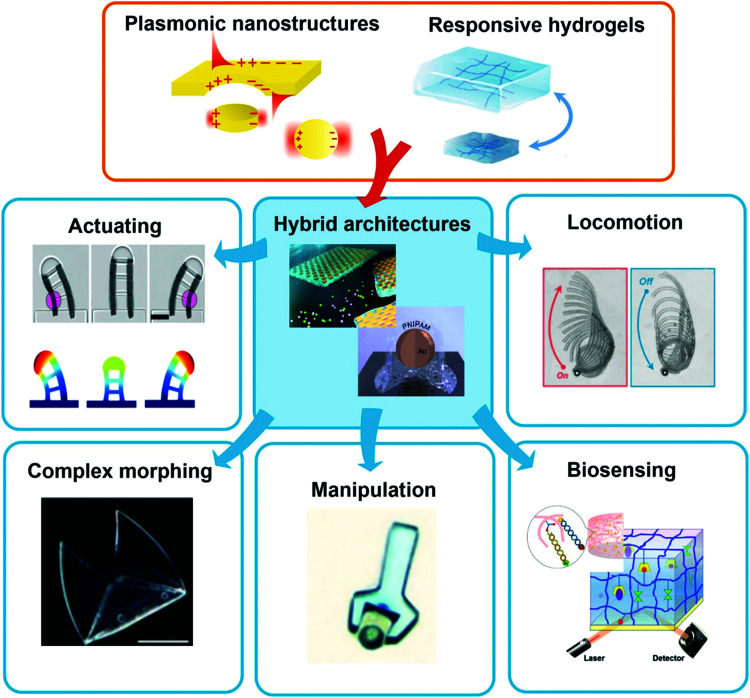
Schematic representation of the combination of plasmonic nanostructures with hydrogels to yield advanced hybrid architectures that can further be employed in the fields of sensors and actuators. All shown examples will be discussed in the corresponding chapters further below, respectively. Adapted under CC-BY from ref. 21–24 and with permissions from ref. 25–27 and 28 ©2020 American Chemical Society, and ref. 29 © IOP Publishing. All rights reserved.

This review highlights current activities in the plasmonic research community devoted to novel functionalities and applications enabled by the marriage of responsive hydrogels with plasmonic metallic nanostructures (Fig. 1). Following a tutorial approach, these two key elements are individually introduced. Starting in Chapter 2, responsive hydrogels, their characteristics, means of preparation, and implementation within miniature devices are discussed. Chapter 3 then focuses on the key rules in designing plasmonic nanostructures, routes for their preparation, and possible strategies for integration into crosslinked polymer networks. Subsequently, novel biosensing modalities that benefit from actively tunable plasmonic materials are presented in Chapter 4, including those with colorimetric and refractometric readout as well as plasmonically amplified fluorescence and surface-enhanced Raman spectroscopy. In particular, we focus on systems in which the open, permeable network structure of the responsive hydrogel is modified with functional biomolecules that specifically interact with the target analyte present in a liquid sample that is contacted with the structure. The capture of target analytes can then serve as an internal stimulus itself. In addition, external triggering of the collapse of the responsive hydrogel matrix with the captured analyte is exploited for compacting the analyte at the so-called plasmonic hotspot. There it can be efficiently probed by the intense surface plasmon field and thus the plasmon-enhanced optical spectroscopy readout can reach the highest sensitivity. Taking the design strategies to the next level in Chapter 5, the concept of exploiting responsive hydrogel plasmonics for actively shape-changing materials is scrutinized and discussed in the context of actuation and locomotion. Here, recent efforts in developing miniature optically-driven machines that can perform mechanical work using plasmonic heating-based actuation are presented. It should be noted that the combination of plasmonic nanomaterials with responsive hydrogels finds also its way to other important applications such as the prominent field of drug delivery^30–32^ that has been covered by recent reviews,^33–35^ but which is beyond the scope of this present review.

## 2. Responsive hydrogels

### 2.1 Definitions: hydrogels

Polymeric gels^36^ play an essential role in the development of innovative soft matter applications due to their unique characteristics, like viscoelasticity and swelling behavior. These polymeric systems are distinguished from inorganic gels, like silica gels consisting of inorganic SiO_2_ particles, which are not in the scope of this review. By definition, a polymeric gel is a highly solvent-swollen polymer network of macromolecular chain segments held together by interconnections, *i.e.* crosslinks, which can either be of chemical or physical nature. The chain segments are characterized by the number of repeat units between two crosslinks, which defines their segment length *l*_s_. The crosslinks prevent the macromolecular system from dissolving as individual chains and allow for a substantial uptake of liquid by chain solvation, resulting in swelling of the gel. Based on this attribute, the volume swelling ratio *Q*_V_ is defined by the quotient of the total volume of the swollen system, including the polymer network and the associated solvent, divided by the volume of only the polymer network, and corresponds directly to the inverse of the polymer volume fraction *Φ*_p_ (symbols are used in analogy to ref. 36). The swelling ratio depends directly on the number of crosslinks per volume element in the polymer network, referred to as crosslink density *ν*_c_. For a given polymer, the swelling ratio *Q*_V_, crosslink density *ν*_c_, and segment length *l*_s_ are interrelated as follows: the higher the crosslink density *ν*_c_, the shorter the segment length *l*_s_, the lower the swelling ratio *Q*_V_, and *vice versa*. If the gel is swollen specifically with water, it is called a hydrogel.^37^ Consequently, the primary requisite for a hydrogel is the strong affinity of the underlying macromolecular structure to water. The required hydrophilicity of the repeat units in the polymer chain is provided by functional moieties with high polarity, such as hydroxyl, amino, carboxyl, ionic, or even zwitterionic groups.^38^ The polymers may be derived from hydrophilic monomers based on synthetic sources (like acrylates,^39^ acrylamides,^40^ 2-oxazolines,^41^ and sulfo- or carboxy-betaines^42^), or originating from natural sources (such as dextrans,^43^ alginates,^44^ polypeptides,^45^ proteins,^46^ hyaluronic acid,^47^ and chitosan^48^). The water-swollen, soft materials with high chemical versatility, which hydrogels represent, can mimic living tissue,^49^ be easily integrated into biological systems,^50^ and serve as a binding matrix material in bioanalytical application technologies as well as artificial muscles in soft robotics.^24,51–53^

### 2.2 Definitions: thermo-, iono-, and pH-responsive hydrogels

**Fig. 2:**
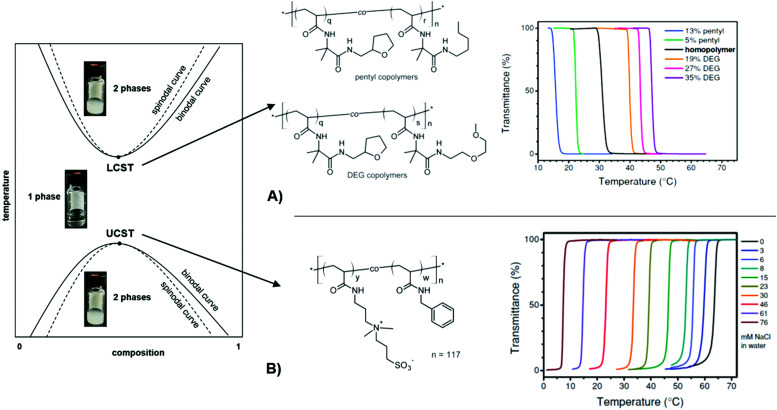
Schematic phase diagram for UCST/LCST-type polymer solutions at different concentrations and temperatures. The photographs visualize the phase transitions of a thermoresponsive polymer in water. (A) Transmittance curves of LCST-type copolymers illustrating the influence of side-chain architecture and compositions on the cloud point. Adapted with permission from ref. 64, ©2013, American Chemical Society. (B) Transmittance curves of a UCST-type, sulfobetaine-based copolymer in water at varying salt concentrations, showing enhanced solubility with an increased salt concentration. Adapted with permission from ref. 65 ©2014, American Chemical Society.

A particularly interesting category of hydrogels draws on the intricate properties of stimuli-responsive polymers that undergo physical transformations induced by an external stimulus, like due to a change in illumination (photoresponsive),^54^ ionic strength (ionoresponsive),^55^ proton concentration (pH-responsive),^56^ or temperature (thermoresponsive).^57^ Such transformations may occur in the form of shape and volume changes, sol–gel transitions, assembling and disassembling, or switching between hydrophobic and hydrophilic interactions.^58^ In order to yield a multiresponsive hydrogel, several polymer classes, which are sensitive to different triggers, may be integrated into the same network structure.^59,60^ In this review, we mainly focus on responsive hydrogels, in which a volume change is triggered by a shift in temperature, light, pH, or salt concentration. The mechanism responsible for volume changes of thermoresponsive hydrogels is found in the temperature-dependent interaction of polymeric substructures with the surrounding solvent. For freely dissolved polymer chains, this transition is manifested as a cloud point temperature (*T*_c_), at which the optical transmittance of the solution is either decreased by precipitation or increased due to dissolution of the polymer (Fig. 2, left). The transition temperature for a given polymer structure specifically depends on the type of solvent (water in the case of hydrogels), the concentration of the solution, and the heating or cooling rate.^61^ There are two general types of transitions with either an abrupt dissolution at the upper critical solution temperature (UCST)^61^ or precipitation at the lower critical solution temperature (LCST)^62^ upon temperature increase. Below the *T*_c_, an LCST-type polymer exists in a solvated state as equilibrated coil. Upon exceeding the *T*_c_ the polymer chains can undergo a coil-to-globule transition, releasing water from the solvation shell, and typically precipitate (if not colloidally stabilized).^63^ For UCST-type polymers, the opposite behavior is observed, being insoluble at low temperatures and dissolving when reaching the *T*_c_. A commonly used technique to determine the *T*_c_ of a polymer solution is turbidimetry. For this method, transmittance of the solution with given concentration is measured while heating or cooling the sample at constant rate. With this method, the influence of several parameters on the transition behavior of responsive polymers was investigated.^64,65^ In an earlier publication, the synthesis of different LCST-type copolymers by postmodification of poly(2-vinyl-4,4-dimethylazlactone)s with appropriate substituents (here: tetrahydrofurfuryl, *n*-pentyl, or di(ethylene glycol) methyl ether side chains) was described to shift the transition temperature.^64^ The dependency of the *T*_c_ on the polymer structure with varying side group type and ratio was demonstrated *via* turbidimetry (Fig. 2A). While the increase in hydrophobicity of the copolymer by the *n*-pentyl side chain leads to a decrease of *T*_c_, the di(ethylene glycol) methyl ether side chains are more hydrophilic and consequently increase the *T*_c_. In a second publication, turbidity measurements of an UCST-type sulfobetaine copolymer at different sodium chloride concentrations (Fig. 2B) are reported.^65^

**Fig. 3:**
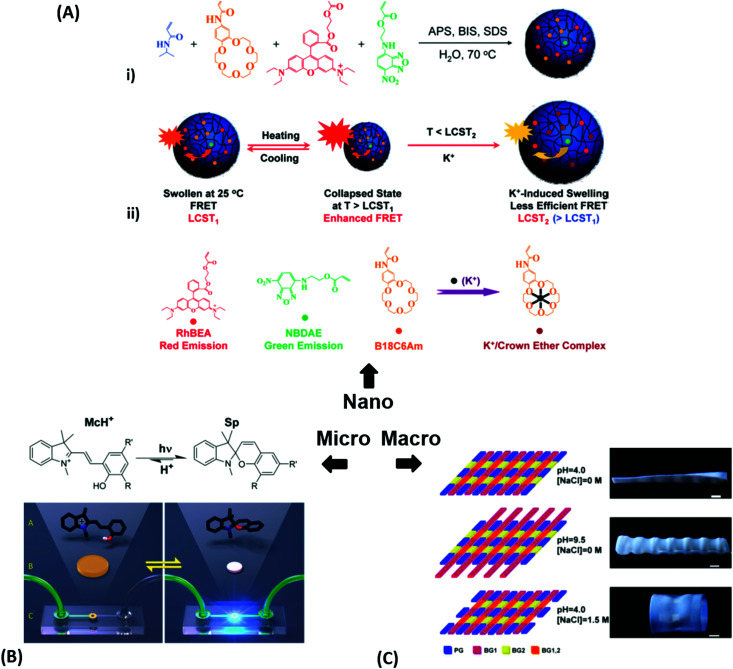
(A) – (i) Synthesis scheme for the preparation of thermoresponsive and K^+^ responsive hydrogel particles in the 100 nm size range. (ii) Illustration of fluorescence resonance energy transfer (FRET) within the hydrogel particles induced by changes in temperature, K^+^ ion concentration, and a combination of both. Adapted with permission from ref. 75 ©2010, American Chemical Society. (B) – Effect of structural isomerization of a photoresponsive molecular probe inside a polymer network on the swelling state of the hydrogel by irradiation with light, which serves as light-switchable valve in a microfluidic device. Adapted with permission from ref. 76 ©2013, American Chemical Society. (C) Multiple shape transformations of pH- and ionoresponsive composite gel sheets. At pH = 4 and [NaCl] = 0, the gel sheet acquires a planar shape. At pH = 9.5 and [NaCl] = 0, the hydrogel forms a long cylinder. At pH = 4 and [NaCl] = 1.5 M, the gel adopts a drum shape. The thickness of the rectangular hydrogel sheet is 0.44 mm and the scale bars are 0.5 cm. Adapted with permission from ref. 60 ©2013, American Chemical Society.

Besides the structural influence of the copolymer composition on the transition temperature, the effect of ion concentration on *T*_c_ is of high importance. As the solubility of the sulfobetaine copolymer is enhanced by the addition of sodium chloride, the *T*_c_ shifts to lower temperatures for higher salt content. With this ionoresponsiveness, it is possible to shift the phase transition to a desired temperature range by adjusting the ion concentration. The same principle is exploited with pH-responsive polymers, which exhibit a particular solubility behavior affected by the proton concentration. For these systems, acidic or basic groups (carboxylic acids, amines, *etc.*) in the polymer structure provide a pH response.^56^ In order to use light as a stimulus, various polymer systems have been realized with photochromic motifs (*e.g.* benzospiran, azobenzene, or triphenylmethane leuconitrile groups).^66–68^ These chromophore moieties undergo a structural change of their molecular framework upon excitation with light (like a *cis*–*trans* isomerization in the azobenzene group or decomposition into an ion pair in the case of triphenylmethane leuconitrile groups). By this, the state of the photosensitive polymer can be reversibly switched, yielding photoresponsive polymer structures. In particular, light features the benefit of being stringently controllable in wavelength, intensity, duration, and spatial resolution. These discussed concepts of tailored thermoresponsiveness, ionioresponsiveness, lightresponsiveness, and pH responsiveness can be transferred from single polymer chains to polymer networks. Since the polymer chains in a network are interconnected, they cannot be completely dissolved. Consequently, for a hydrogel composed of crosslinked thermoresponsive polymers, the temperature-induced transition between a swollen and a collapsed state of the chain segments at *T*_c_ leads to a macroscopic volume change by incorporation or expulsion of solvent from the hydrogel matrix, which has been exploited in a broad range of applications. For example, the adaptable responsiveness and the switchable permeability of the water-swollen hydrogel matrix is appealing for biomedical applications like drug delivery.^69^ In this context, both the temperature and pH sensitivity of responsive hydrogels are exploited for the release of loaded drug cargo at a particular temperature (*e.g.* at human body temperature),^70^ and pH values of the target milieu (*e.g.* blood, stomach, or intestine).^71^ In case of a polymer network scaffold built from photoresponsive polymer chains, the light-induced molecular transitions can be exploited to switch between different states, like closed and open valves,^72,73^ or fouling and antifouling surfaces for so-called “self-cleaning” purposes.^74^ In general, stimulus-triggered response affects the material properties at different length scales and thus allows to exploit these transitions in a variety of functionalities in microscopic as well as macroscopic devices. This is demonstrated in Fig. 3 by characteristic examples of actuators and sensor systems that take advantage of (multi-)responsive hydrogels.

At the nanoscopic scale, the combination of thermoresponsiveness and ionoresponsiveness was demonstrated for dual responsive hydrogel spheres with diameters around 100 nm, which is shown in Fig. 3A.^75^ These systems were utilized as K^+^ sensors by visualizing changes in the fluorescence resonance energy transfer (FRET) intensity that are caused by particle swelling and contracting upon variation of the ion concentration and temperature. Such swelling-dependent sensing can as well be achieved by exploiting the plasmonic response of small hydrogel beads with incorporated gold nanostructures. These plasmonic systems respond chain segment elongation inside the polymeric network leading to the modulation in near-field optical coupling that can be perceived as a color change, as discussed in the corresponding chapters below in more detail.^77^ In the microscopic domain, there was demonstrated the application of volume-changing hydrogels in microfluidic valves that can be opened and closed by a light stimulus (Fig. 3B).^76^ A macroscopic example was provided with composite gel sheets exhibiting a size in the cm range, which show dual-responsiveness towards pH and ion concentration by reversible folding and stretching in opposite directions (Fig. 3C).^60^ Based on the complex stimuli-induced shape change, self-folding tubes were prepared that change diameter and length in response to the solute composition in the liquid medium. These three examples provide only a brief glimpse into the limitless possibilities and the immense potential that multiresponsive hydrogel systems may offer to soft matter research. In order to tap into this rich arsenal of polymer-based hydrogel materials, first, their synthetic fundamentals need to be embraced.

### 2.3 Hydrogel preparation: polymerization and crosslinking

**Fig. 4:**
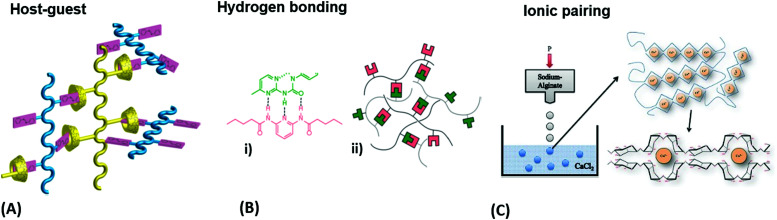
(A) Schematic illustration of a host-guest network with physical crosslinks from a mixture of azobenzene copolymers and α-cyclodextrin copolymers. Adapted with permission from ref. 80. (B) Exploitation of hydrogen bonding motifs for multivalent crosslinking of macromonomers: (i) the underlying chemical complex and (ii) schematic representation of the hydrogen-bonded network. Adapted with permission from ref. 81. (Further permissions related to the material excerpted should be directed to the ACS.) (C) Gelation process of alginate by crosslinking of the polysaccharide chains with calcium ions after injecting sodium alginate into a calcium chloride solution. Adapted under CC-BY 3.0 from ref. 83.

Water-attracting interconnected polymer chains are the prerequisite for hydrogels and the following paragraph expatiates their most common synthesis considerations and crosslinking strategies. The hydrophilic polymer chains are formed by common polymerization methods,^78^ like free radical polymerization, ring-opening polymerization, or polycondensation, in which the crosslinks can be introduced either during or after chain formation, as specified further below. Crosslinks can be of chemical nature, like permanent covalent bonds, or they are based on reversible physical interactions, such as entanglements, host-guest interactions, hydrogen bonding, ion-pairing, or van der waals forces.^79^ Entanglements affect the polymer behavior once the chain exceeds a critical length that is about twice the segment length *l*_s_ between two entanglements.^78^ Host-guest systems involve the complexation of a molecular entity (guest) within a molecular cavity (host), as given for the azobenzene guest inserted into a cyclodextrin host, depicted in Fig. 4A.^80^ Hydrogen bonding typically exists between polar groups with high electron density (*e.g.* carbonyl oxygen), so-called acceptors, and groups with a labile bond to a hydrogen (*e.g.* amide proton), referred to as donor (Fig. 4B).^81^ Such hydrogen bonding motives acting as crosslinks can either be intentionally integrated into the polymer backbone during synthesis *via* the introduction of appropriate functional groups or be intrinsically present in certain polymer classes, like in gelatin.^82^ Depending on the solvent and the type of ionic interaction, ion-pairing may lead to a physical network, as illustrated by the example of calcium-induced crosslinking of sodium alginate (Fig. 4C),^83^ where the exchange of the monovalent sodium ions with the divalent calcium ions transforms the soluble polymer into a hydrogel by ion bridging.

The strategies mentioned above, yielding reversible, physical crosslinks are suitable for applications, in which transient networks and temporary gel formation are desired. Such reversible network junctions provide a responsive structure component for reconfiguration of the gel scaffold *via* the action of an external stimulus. By opening or closing the network junctions, the crosslink density *ν*_c_ is varied, which has a direct influence on the mechanical properties and the swelling behavior. This responsiveness can be exploited for mechanical work in actuator applications or as transducer elements in gel-based biosensors.^84^ However, often enhanced stability and durability of the hydrogel scaffold are required, demanding permanent covalent network junctions. Depending on the target hydrogel structure and application, chemical crosslinks can be introduced during (*in situ*) or after polymerization (post-synthetic).^38^ For *in situ* crosslinking, multifunctional crosslinkers (monomers that can engage in more than two covalent bonds) are employed as comonomers in the polymerization reaction, which yields branches and crosslinks directly. In contrast, the post-synthetic approach requires an additional reaction step after polymerization to activate the crosslinker to interconnect the polymer chains. Such crosslinker functionality can be integrated into the polymer backbone during polymerization by a comonomer with a dormant reactive site (here referred to as internal crosslinker), which can be independently activated to form covalent bonds with neighboring chains. Alternatively, a polymer with appropriate functional groups can be reacted by addition of a small molecular agent (here referred to as external crosslinker) to generate the network in the post-synthetic step. Consequently, internal crosslinkers exhibit one chain-extending unit (*e.g.* double bond for polymerization) and at least one crosslinking unit (introducing a branching point for chain interconnection), while external crosslinkers should carry at least two interconnecting units. The crosslinkers for post-synthetic network formation may be activated by irradiation with light, by temperature increase, or by other stimuli. An external crosslinker is further required to be well miscible with the polymer matrix in order to prevent phase separation of the system. Internal crosslinking can be performed in two different ways following distinct generic mechanisms, either by specific pairing of complementary functional groups or by unspecific covalent linkage with the surrounding molecular framework. In the first case, the internal crosslinkers undergo specific reactions, like [2+2]-cycloaddition (Diels-Alder reaction),^85^ Michael addition (“click-chemistry”),^86^ or 1,3-cycloaddition.^38^ The advantage of this specific post-synthetic crosslinking method lies in the predictability of the target structure for the network junctions. However, if the concentration and the mobility of the specific crosslinker units are too low, the probability for an encounter to form the covalent network link by reaction is strongly reduced.^87^ In the second case, unspecific crosslinker units (*e.g.*, aromatic ketones,^88^ azides,^87^ or diazo groups^85^) may react by an unspecific C–H insertion reaction after activation by heat or light. As an advantage, such unspecific crosslinkers can act at very low concentrations as they do not require a specific reaction partner but rather undergo covalent linkage with either polymer backbones or side chains. Even more so, they can induce concurrent anchoring to a suitable substrate carrying the required C–H groups.^41,85,89^ This simultaneous crosslinking and surface-attachment strategy is of great interest for the fabrication of sensors, actuators, and antifouling surface coatings.^18^ Furthermore, the above outlined strategy to activate the post-synthetic crosslinker by irradiation with light can be conveniently exploited to spatially address network formation to yield advanced constructs with predetermined 2D patterns and 3D geometries, as described further below.

### 2.4 Hydrogel characteristics: tailoring of properties

In order to provide the most suitable features of a hydrogel for the specifically targeted application, its characteristic properties can be tailored by the following four aspects.

#### 1. Molecular basis

The specific chemical structure and dedicated functional groups of the underlying monomeric building blocks, as well as their combination in copolymers, provide the foundation for the desired hydrogel characteristics. For example, the monomer *N*-isopropylacrylamide (NIPAAm) was used to introduce the thermoresponsiveness in LCST-type hydrogels.^90–92^ Equally, highly hydrophilic betaine monomers were employed in polymers to implement a UCST response and potentially resistance against unspecific sorption of biomolecules from complex biological fluids.^42^ Such antifouling properties were exploited in applications, where biomolecular adhesion is not desired, like on ship hull surfaces,^93^ medical devices and implants,^94^ or biosensor chip interfaces.^42^

#### 2. Network design

The crosslink density *ν*_c_ influences the degree of swelling and associated mechanical and diffusive properties. A higher number of crosslinks reduces the swelling capacity and consequently increases the stiffness of the gel. Inversely, a lower crosslink density *ν*_c_ entails a higher swelling ratio *Q*_V_ and the associated larger, solvent-filled voids between the polymer segments allow an enhanced diffusion of small solute molecules through the network structure.

#### 3. Network modification

The material properties of the network can be further tuned by post-synthetic functionalization or by blending with other constituents to form a hybrid or composite material. Such functionalization strategies may employ integrated carboxyl groups for subsequent conjugation with biomolecules such as immunoglobulin G antibodies,^91^ or with other specific biological receptors^95,96^*via* amine coupling. Furthermore, the inclusion of nanoparticles allows targeting special properties of the hydrogel composite system,^97^ like plasmonic heating *via* embedded gold nanoparticles,^92^ mechanical reinforcement,^98^ or magnetic manipulation.^99^

#### 4. Architectural aspect

**Fig. 5:**
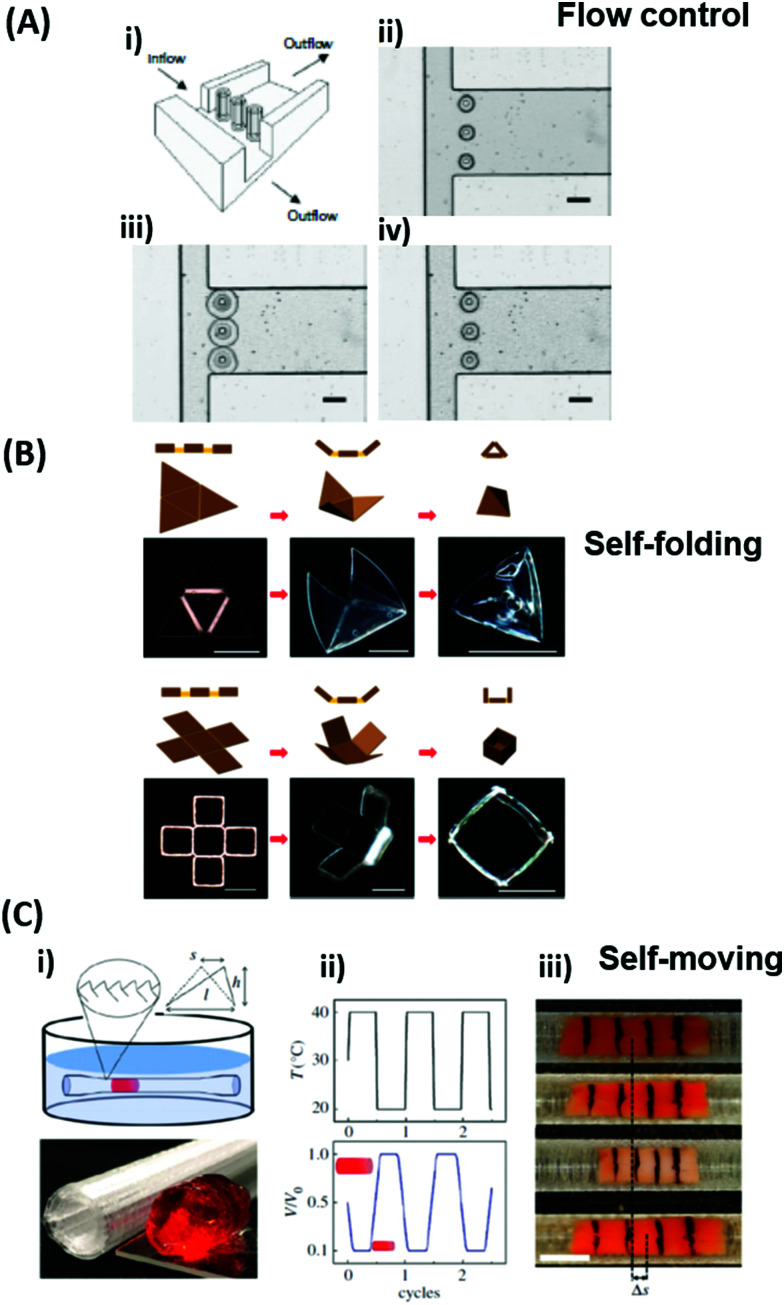
(A) Temperature-switchable valve structures based on rigid posts in a microchannel coated with thermoresponsive hydrogels. (i) Model of the flow regulation system. (ii) Optical micrograph of the actual device in the open state after polymerization of the hydrogel. (iii) In the closed valve state the swollen hydrogel jackets block the side channel. (iv) Reverted to the open valve state the contracted hydrogels allow fluid to flow down the side branch. Scale bars, 300 μm. Adapted by permission from Springer Nature from ref. 99 ©2000. (B) Schematic and optical microscopy images showing thermally responsive self-folding hydrogel structures of different shapes, such as a pyramidal and a cubic capsule. The optical images were taken while increasing the temperature from 25 °C to 60 °C. All scale bars are 300 μm. Adapted with permission from ref. 108 © IOP Publishing. All rights reserved. (C) Temperature-driven pNIPAAm-based hydrogel crawler. (i) Schematic of the experimental set-up. (ii) Schematic plots showing the periodic change of temperature of the fluid and the resultant swelling/collapsing of the hydrogel. (iii) Sequential snapshots of the hydrogel in one collapsing/swelling cycle in the channel. Scale bar: 1 cm. Adapted with permission from ref. 105.

The hydrogel material can be furthermore sculptured to adopt complex shapes and structures at length scales from the nanoscopic level up to macroscopic dimensions. The methods to precisely control the geometry and dimensionality of the hydrogel structures can be chosen from a large pool of patterning techniques, which are covered further below. The generic structure classes can be distinguished as bulk hydrogel, micro or nanogel particles,^100^ surface-attached hydrogel layers,^87^ and polymer brushes.^101^ While the unconfined gel material in the bulk or particle form can freely swell in three dimensions, confined networks can only swell with reduced dimensionality. These include 2D swelling in the valve structures shown in Fig. 5,^99^ or 1D swelling in surface-attached hydrogels and polymer brushes deployed at planar substrates, where swelling occurs primarily in the direction perpendicular to the surface.^38^ The concept of 1D swelling hydrogels is of high relevance for antifouling coatings,^69^ sensing,^20,102,103^ and actuation applications.^104,105^ Strictly speaking, polymer brushes do not represent a true category of hydrogel networks since the main feature of a hydrogel – the presence of crosslinks interconnecting the polymer chains – is missing. However, they exhibit similar properties like surface-attached polymer networks, and the brush layers can be produced with crosslinks, which are termed brush-gels.^106,107^

Since many strategies are available to tailor hydrogel properties towards a target application, their combination leads to an even larger variety of achievable features, as demonstrated with the following three examples. In the earlier developments, there was pursued pH-responsive valve based on functional hydrogel architecture of hydrogel layers attached to supporting posts that were prepared inside a microfluidic device (Fig. 5A).^99^ For this purpose, a mixture of acrylic acid (for pH response) and 2-hydroxyethyl methacrylate (HEMA, hydrophilic comonomer for water uptake) was polymerized using the *in situ* crosslinker ethylene glycol dimethacrylate in the presence of a photoinitiator and with the help of a photomask. The modified microfluidic device allowed flow control of liquid through the side channel by the pH-responsive valve, which could collapse or swell within seconds upon pH change. A similar flow regulation system based on light-induced plasmonic heating of optically independently addressable gold colloids and nanoshells to trigger a thermoresponsive hydrogel collapse was exemplarily introduced.^109^ A later report was published on self-folding 3D hydrogel scaffolds with micrometer dimensions, which were pH- and thermoresponsive. There were lithographically inscribed zones with different crosslink density *ν*_c_ and gradients into the hydrogel network to exploit the resulting difference in mechanical properties and swelling behavior as rigid walls and flexible hinges (targeted network design, Fig. 5B).^108^ The utilized photoreactive hydrogel precursor was composed of an *n*-butanol solution of thermoresponsive LSCT-type p(NIPAAm), NIPAAm monomer, pH-responsive acrylic acid, the *in situ* crosslinker *N*,*N*′-methylenebisacrylamide, and a photoinitiator. Structural elements of the 3D scaffold were generated from the hydrogel precursor by irradiation through a photomask. The highly swellable hinges were built by thin, gradiently crosslinked regions, while the rigid, low swelling panels were made from thick sections with high crosslink densities. When immersing these geometric hydrogel structures in water and increasing the temperature over the *T*_c_ of pNIPAAm, the temperature-induced self-folding of the 3D scaffold took place (see Fig. 5B). In a more recent example, self-crawling hydrogel particles are reported that move inside a surface-modified channel upon periodical temperature changes, falling below or exceeding *T*_c_.^105^ Their actuation system was based on thermoresponsive LCST-type hydrogels formed by pNIPAAm chains, confined in a narrow, cylindrical channel with rough, anisotropic surface structure (Fig. 5C, left). The NIPAAm-based hydrogel corpus collapsed, when immersing this system into an aqueous medium with a temperature below *T*_c_, and expanded, after immersion into a water bath tempered above *T*_c_. A periodical decrease/increase in temperature resulted in an expansion/shrinkage of the hydrogel inside the channel and a longitudinal movement due to the friction between the hydrogel and the anisotropic surface of the tube (Fig. 5C, right). The main underlying concepts for hydrogel crawlers are discussed in more detail in the later chapter dedicated to hydrogel actuators. Even more complex functionality can be achieved with these responsive hydrogel systems by integration into sophisticated architectures described in the following section.

### 2.5 Structured hydrogels: realization of complex architectures

**Fig. 6:**
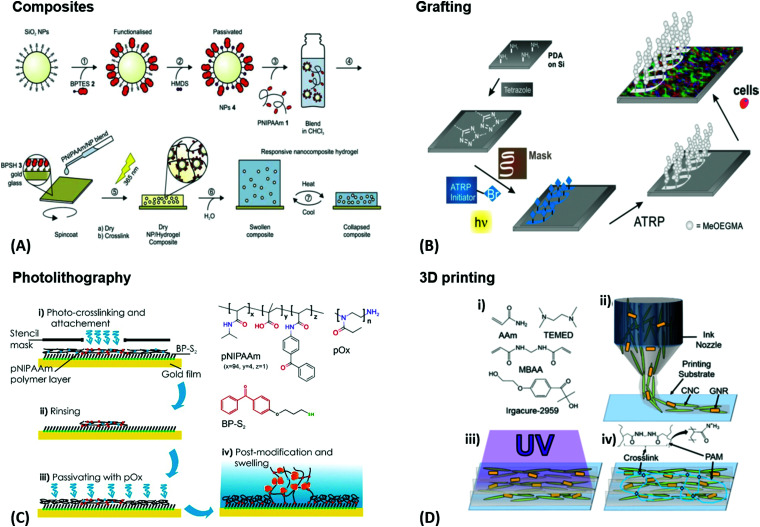
(A) Preparation of photoreactive functionalized SiO_2_ nanoparticles and their application in responsive nanocomposite hydrogels for mechanical stabilization. Adapted with permission from ref. 98. (B) Surface patterning of silicon wafers and their resistance to cell adhesion. A film was functionalized with a photoactive tetrazole. A maleimide-based initiator for atom transfer radical polymerization was photopatterned *via* irradiation through a photomask. Polymer brushes were then grown on the patterned areas, showing full resistance towards cell adhesion while the rest of the surface was coated by a confluent layer of cells. Adapted with permission from ref. 111. (C) Application example for the photolithography process. A photocrosslinkable polymer is spin coated and irradiated with UV light through a stencil mask. Non-irradiated (and therefore non-crosslinked) areas are washed off. An additional passivation step may be implemented as well. (D) – (i) Underlying chemical structures that are mixed with gold nanorods and cellulose nanocrystals to make the gel precursor. (ii) Cellulose nanocrystals and gold nanorods are orientationally aligned along the flow of the gel precursor by a 3D printing process. (iii) Curing of the printed sol with UV light. (iv) Resulting composite gel with crosslinked poly(acrylamide) chains. Adapted with permission from ref. 112. (Further permissions related to the material excerpted should be directed to the ACS.)

An intrinsic structuring can be found in composite hydrogel materials obtained by modification of polymeric gels with filler components, like fibers or nanoparticles. Depending on the properties of the individual components, this approach can result in responsive composites with complex architectures. Such hydrogel composites can be prepared by three strategies, being (1) addition of the respective filler component to the pre-formed hydrogel matrix, (2) mixing of the filler with a polymer solution prior to the polymer crosslinking, or (3) polymerization of the hydrogel-forming monomer in the presence of filler particles and fibers.^97^ For example, such composite materials can provide increased mechanical stability compared to the parent hydrogel (Fig. 6A). In this approach, recent work exploited silica nanoparticles that were integrated into the thermoresponsive pNIPAAm network in order to reinforce the soft hydrogel matrix.^98^ In general, the sophisticated combination of the specific filler properties with the responsiveness of the polymer network can be employed as stimulus transducer, for example a hydrogel collapse induced by plasmonic heating *via* optical irradiation of embedded gold nanoparticles. As stimulus transducers are essential components of sensor systems, these responsive hydrogel composites may be directly applied as sensing matrices. One of the most common architectures in sensor and actuator platforms consists of a thin hydrogel layer covalently attached to a surface, which shows 1D swelling perpendicular to the surface. The surface acts as solid support and can simultaneously be involved in the transduction and sensing process, which is exploited with the plasmonic response of gold surfaces. Depending on the actual hydrogel layer thickness, different preparation methods have to be employed. For ultra-thin polymer brush layers with the thickness defined by a single polymer chain, two general synthesis techniques are employed, referred to as “grafting-to” and “grafting-from” method.^42^ The grafting-from method allows the formation of attached polymer chains by polymerizing the monomers away from the surface starting at a surface-attached initiator species, while grafting-to refers to the deposition of the preformed polymer chains. These polymer brush layers can also be laterally patterned, as outlined in Fig. 6B. For preparation of thicker hydrogel films, doctor blading, printing, spin coating, dip coating, or spray coating of the polymeric precursor can be employed, followed by crosslinking activated with an external stimulus (*e.g.* heat or light).^110^ Often, a covalent surface attachment of the hydrogel network is simultaneously achieved in this crosslinking step. Furthermore, hydrogel layers may be prepared by an assembly of pre-formed hydrogel beads (micro- and nanogels) on the substrate surface. A standard deposition method is the Langmuir–Blodgett technique, in which the particles are transferred to a solid substrate by its slow withdrawing through an air-liquid interface, at which the particles self-assemble.

For precursor polymers with photosensitive crosslinker units, photolithography can be utilized to directly generate intricate patterns of small hydrogel structures by light-induced crosslinking. The required spatially controlled irradiation can be achieved by either illumination through a photomask or by a focused optical beam. By the first method, only areas irradiated by UV light are photocrosslinked, whereas the polymer chains on the surface area protected by the lithography mask stay unreacted and can be washed away after the process, as presented in Fig. 6C. The latter one, two-photon polymerization of hydrogel precursors, represents a more advanced technique to fabricate 3D hydrogel structures by direct laser writing with high resolution below the 100 nm limit and structure dimensions up to macroscopic length scales, also referred to as nanoscale 3D printing.^113–116^ In order to achieve rapid fabrication of macroscopic hydrogel scaffolds and objects, various 3D printing technologies are available for fully automated printing of a precursor polymer with subsequent crosslinking. An example of this technique was demonstrated to yield well-defined poly(acrylamide) composites with co-aligned cellulose nanocrystals and plasmonic gold nanorods, as shown in Fig. 6D.^112^ In 4D printing, the above-mentioned structuring tool provided by 3D printing is combined with the unique properties of responsive hydrogel materials to transform the shape of the object by application of an appropriate stimulus after printing. In the following chapters, the combination of responsive hydrogel materials and plasmonic structures is highlighted with recent examples of sensor and actuator applications.

## 3. Responsive plasmonic nanomaterials

Metallic nanoparticles and nanostructured metallic thin films increasingly serve as building blocks for the assembly of materials with precisely tailored optical properties. These structures enable to tightly confine energy of light at their surface by the resonant excitation of surface plasmons that originate from coupled collective oscillations of electron density and associated electromagnetic field. The combination of metallic nanostructures with responsive hydrogels paved the way to numerous important features and functionalities, including facile means to modify metallic surfaces with functional biomolecules (for biosensor applications), ‘on-demand’ tuning their optical properties (serving in enhanced optical spectroscopy and adaptive optical materials), as well as performing light-fueled mechanical work and actuating (in micro- and nano-machines).

### 3.1 Optical properties of plasmonic nanostructures

**Fig. 7:**
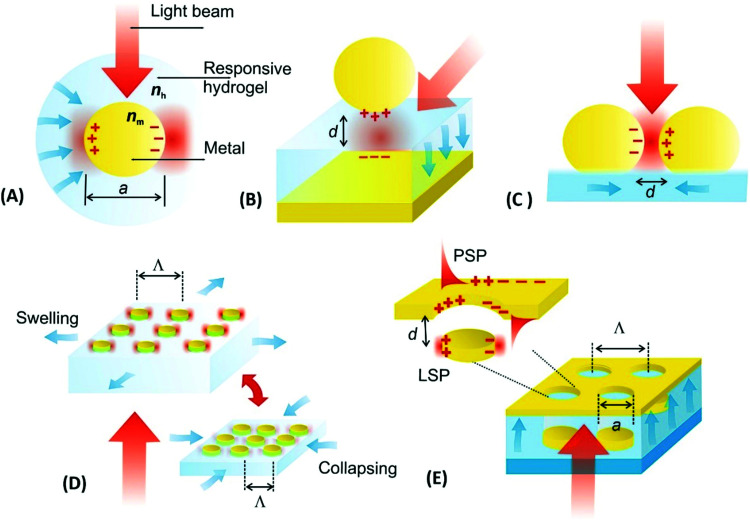
Overview of actuated geometries supporting localized surface plasmon (LSP) and propagating surface plasmon (PSP) modes by the use of responsive hydrogels: (A) individual metallic nanoparticle responding to changes in refractive index *n*_h_ of the hydrogel capping shell, (B) metallic nanoparticle placed at tunable distance *d* from a flat metallic surface, (C) plasmonic nanoparticle dimer with actuated separation distance *d*, (D) lattice of metallic nanoparticles with modulated period *Λ* and (E) example more complex structure where responsive hydrogel induced variations in both refractive index *n*_h_ and near-field coupling responding to *d*.

The optical properties of metallic nanostructures and architectures they constitute can be efficiently controlled by their architecture design. The particular parameters that allow tuning such characteristics include the shape and material from which the individual metallic nanoparticles are made, their spatial arrangement, and optical properties of the surrounding dielectric. In general, the use of responsive hydrogels as materials hosting the metallic nanostructures enables ‘on demand’ controlling the latter two parameters, which open doors for developing new class materials that exhibit actively tunable characteristics. As illustrated in Fig. 7, the swelling and collapsing of the responsive hydrogel matrix can be utilized to reversibly modulate refractive index *n*_h_ (due to the variations in polymer volume fraction *Φ*_p_) and the distance between metallic nanoparticles *d* (through volumetric change of the polymer networks). These parameters thus serve as facile handles to actuate the resonant coupling of light to localized surface plasmons (LSPs) supported by metallic nanoparticles and propagating surface plasmons (PSPs) that travel along the continuous metallic films.

The first approach presented in Fig. 7A shows actuating of LSPs supported by an individual metallic nanoparticle by changing the refractive index *n*_h_ of the surrounding responsive hydrogel shell. In general, collapsing of the hydrogel shell adjacent to the metallic core leads to a local increase in the refractive index *n*_h_ that red-shifts the resonant excitation of LSPs to longer wavelengths. For instance, a collapse of about 100 nm thick pNIPAAm-based hydrogel shell was exploited for detuning the LSPs on chemically synthesized spherical gold nanoparticles with a diameter of 15–50 nm in dynamic light scattering studies.^92^ A higher sensitivity of the LSPs to refractive index changes can be achieved for other shapes of metallic nanoparticles as was, for instance, shown for chemically synthesized rod-shaped gold nanoparticles with pNIPAAm shell.^117^ Alternatively, lithography allows for the preparation of controlled shape of metallic nanoparticles at a solid surface and, for example, arrays of cylindrical gold nanoparticles were fabricated and wrapped by pNIPAAm-based hydrogel caps for their rapid actuating by applied external stimulus.^118^

Metallic nanoparticles separated by a responsive hydrogel from a flat metal surface constitute the second widely used design of tunable plasmonic geometry, see Fig. 7B. It allows for efficient actuation of LSPs that form a plasmonic mode which tightly confines the energy of light in the gap. This gap is defined by the distance *d* between the surfaces of the metal film and the nanoparticle occupied by the responsive hydrogel material. In this architecture, a responsive pNIPAAm-based hydrogel spacer was demonstrated to enable thermal reversible modulating the distance *d* between 18 and 63 nm for gold spherical with a diameter of 78 nm.^119^ In order to actuate narrower gaps, pNIPAAm or pDEAEMA brushes^120,121^ can be tethered to a flat metal surface to serve as a tunable spacer instead of polymer networks forming a thin hydrogel. Moreover, chemically synthesized gold nanoparticles were capped with pNIPAAm polymer and attached to a flat gold surface with the actuated distance *d* between 8 and 20 nm.^122^

Metallic nanoparticle dimers that are sketched in Fig. 7C, or more generally, aggregates of multiple plasmonic nanoparticles, represent another system for tuning LSPs by near-field coupling. For these architectures, the observed LSP resonance is red-shifted to a longer wavelength when reducing the distance *d* between the metallic nanoparticles to comparable or smaller separation than their size *a*. This shift originates from the interaction of LSPs supported by the individual nanoparticles, leading to the occurrence of a LSP mode that also confines the energy in the gap. Such LSP coupling has been implemented with metallic nanoparticles attached to the surface of responsive microgels, that are pushed closer to each other upon the collapse of the responsive microgel core.^123^ For gold nanorods attached to pNIPAAm microgel with a diameter of ∼500 nm, a reversible LSPR wavelength shift of Δ*λ* > 100 nm was observed.^124^ Another possible utilization of this concept was explored by loading chemically synthesized silver nanospheres inside a pNIPAAm microgel body that was made reversibly collapsing and swelling upon temperature modulation.^125^ Morover, an aggregation of gold chemically synthesized nanoparticles capped with pNIPAAm polymer shell allowed for reversible assembly and disassembly of clusters exhibiting distinct plasmonic properties.^126^

**Fig. 8:**
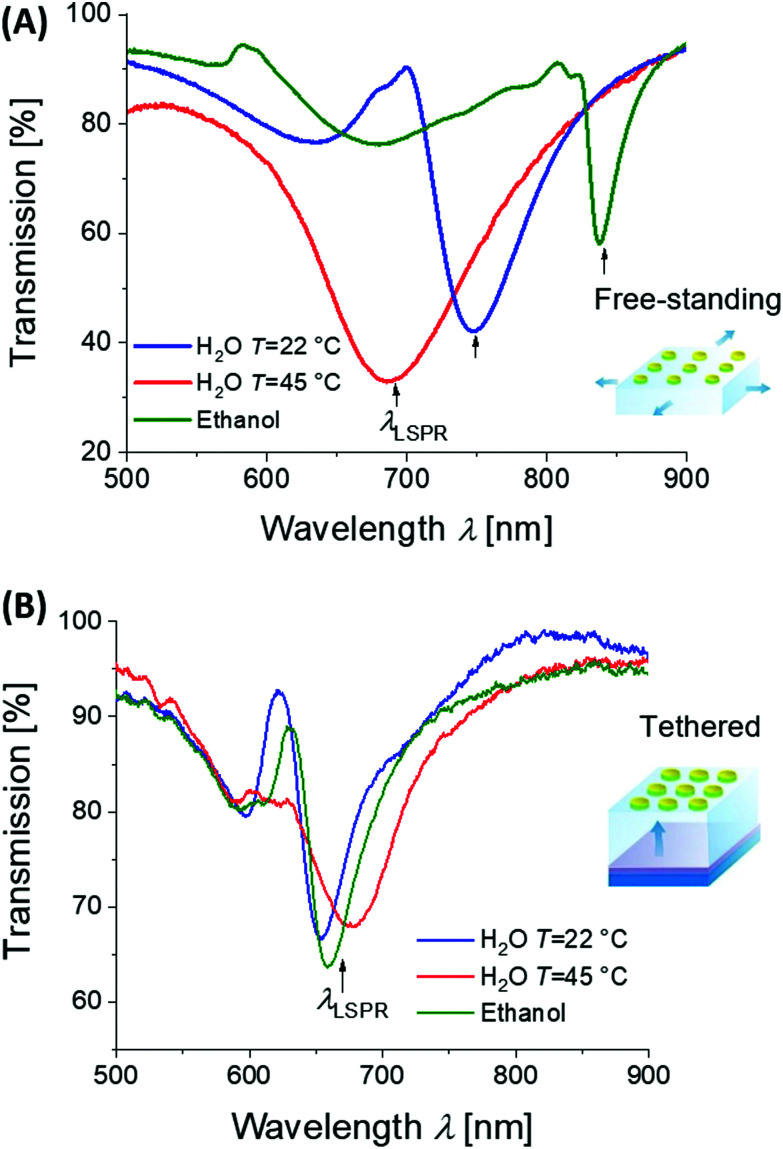
Example of plasmonic properties of lithographically prepared arrays of cylindrical gold nanoparticles on top of a thin pNIPAAm-based hydrogel cushion. (A) Optical response to variations in the period of the structure *Λ* with free-standing membrane, (B) optical response to refractive index changes for the surface-attached structure. Adapted under CC-BY from ref. 128.

By lithography approaches, more precise control of the prepared geometry can be achieved. As Fig. 7D illustrates, two-dimensional periodic arrays of metallic nanoparticles can be prepared for dynamic tuning of LSPs by modulating the period *Λ*. This type of structure was realized with a poly(acrylic acid) (pAAc) hydrogel membrane that carried rectangular gold nanoparticles fabricated by electron beam lithography (EBL).^127^ It exhibited a particle-particle gap of 15 nm that was much narrower than the period *Λ* of about 100 nm. By swelling and collapsing of the pAAc membrane due to the ionic strength variations, the period *Λ* was modulated between 100 nm and 200 nm. These changes lead to the variations in near-field coupling between the LSPs on neighboring nanoparticles, and decreasing the period *Λ* was accompanied by a strong red-shift in the LSP wavelength of Δ*λ* = 70 nm. A similar type of structure was obtained by UV-laser interference lithography (UV-LIL) on the top of a free-standing hydrogel membrane based on pNIPAAm, featuring a longer period *Λ* and wider gap between the metallic nanoparticles of several hundreds of nanometers. Swelling and collapsing of the hydrogel allowed changing the arrays period *Λ* between 360 and 600 nm. In this range, the neighboring LSPs interact *via* diffraction, and thus, opposite evolution of the occurs and LSP wavelength blue-shifts (with maximum change of about Δ*λ* ∼ 150 nm) when decreasing the period *Λ* (see Fig. 8A).^128^

It should be noted that more complex geometries allow for modulation of a richer spectrum of surface plasmon modes by hydrogel actuation, as indicated in Fig. 7E. This schematic shows a metallic film perforated by an array of nanoholes that are contacted with an array of metallic nanoparticles. These nanoparticles are separated from the holes by a distance *d* controlled by swelling and collapsing of the responsive hydrogel cushion. The spectral response of series of resonances associated with the excitation of LSPs (confined at the nanoparticles and the nanoholes) and PSPs (traveling along the bottom and top metal film interface) were investigated.^129^ The observed variations in spectral features encoded to the reflected and transmitted waves were ascribed to the competing effects of near-field coupling (driven by variations in *d*) and changes in the refractive index of the responsive hydrogel *n*_h_ (induced by variations in the hydrogel swelling ratio *Q*_v_).

Moreover, hydrogels exhibit a refractive index *n*_h_ that is close to that of water and thus they allow for embedding the metallic nanoparticles in a refractive index-symmetrical geometry when exposed to acqueous environment. Arranging them in sparse arrays then enables efficiently decreasing the damping of LSP resonances. This phenomenon occurs due to phase-matching of light that is resonantly scattered at the periodic arrays of metallic nanoparticle leading to establishing a new type of lattice (also called collective) LSP modes.^130^ As illustrated in Fig. 8A and B, the collective LSPs excitation can occur on periodic arrays of cylindrically shaped gold nanoparticles attached to a swollen hydrogel membrane in contact with water. It is manifested as an angular dispersive dip in the transmission spectrum that is spectrally narrower than that for conventional LSPs excited on gold nanoparticles attached to a substrate with a higher refractive index. The excitation of these two types of LSP modes can be reversibly switched by collapse and swelling of the hydrogel, accompanied by a modulation of the refractive index *n*_h_ (thus perturbing and re-establishing the symmetry). Such behavior was observed for the metallic structures prepared on top of a responsive pNIPAAm-based hydrogel cushion,^128^ as well as for self-assembled architectures with synthetically prepared gold/pNIPAAm hydrogel core/shell nanoparticles.^131^

### 3.2 Preparation of architectures composed of plasmonic nanostructures and responsive hydrogels

As expounded further below, several distinct types of approaches can be identified for the preparation of materials that combine metallic nanostructures and responsive hydrogels. Typically, metallic nanoparticles are fabricated separately and then either embedded into hydrogel materials, attached to their surface, or caped with a responsive polymer network shell for subsequent assembly into the desired composite architectures.

**Fig. 9:**
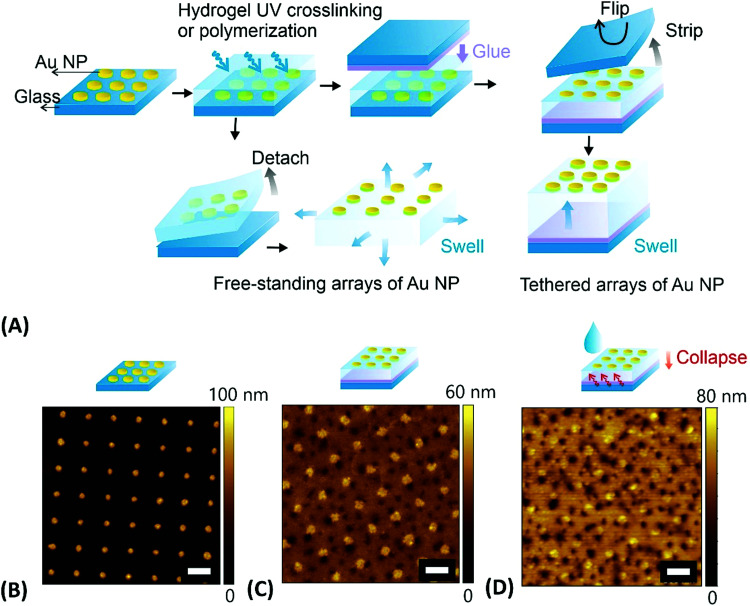
(A) Overview of the preparation steps for embedding gold nanoparticles arrays into tethered and free-standing pNIPAAm-based hydrogel films. AFM observation of (B) the gold nanoparticle arrays prepared by UV-LIL, (C) stripped structure that was attached to the crosslinked pNIPAAm polymer in the dry state in air, (D) same structure under water at a temperature above the LCST. Scale bars correspond to 500 nm. Adapted under CC-BY from ref. 128.

In the first fabrication approach to yield extended arrays of hydrogel-embedded metallic nanoparticles, the metallic nanostructures can be prepared on a solid substrate by using lithography followed by their transfer to the responsive hydrogel material. A wide range of lithography methods has been established for the preparation of such metallic nanostructures, including EBL,^132^ UV-LIL,^133^ and colloidal lithography.^11^ As illustrated in the example presented in Fig. 9, periodic arrays of cylindrical gold nanoparticles were prepared on a glass substrate by a UV-LIL method. Afterwards, these nanoparticles were attached to a NIPAAm-based polymer network in order to obtain thin responsive plasmonic membranes.^128^ This polymer network was prepared either by irradiation of a photocrosslinkable pNIPAAm-based copolymer carrying benzophenone groups or by direct polymerization of monomers in contact with the surface carrying metallic nanostructures. The first route yielded a hydrogel film with a thickness of 2.2 μm, while the second route allowed a significant increase of the attainable thickness to about 40 μm (both in swollen state). Such hydrogel membranes with embedded metallic nanoparticles were then stripped from the substrate to be used as surface-attached or a free-standing structure (see Fig. 9).

**Fig. 10:**
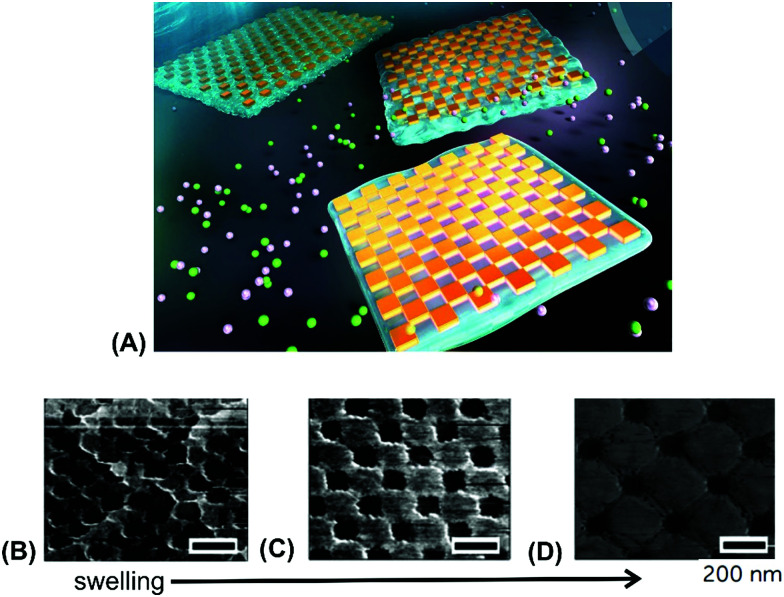
(A) Illustration of a free-standing pAAc membrane with embedded arrays of rectangular gold nanoparticles prepared by EBL. (B)–(D) AFM images of the structures upon gradual swelling. Scale bars correspond to 200 nm. Adapted under CC-BY 3.0 from ref. 127.

A similar strategy was utilized for embedding periodic arrays of rectangular gold nanoparticles into a free-standing pAAc hydrogel membrane (Fig. 10A).^127^ The closely packed rectangular gold nanoparticles were prepared on a Si substrate by EBL and then transferred onto a thin pAAc hydrogel membrane that was allowed to swell in an aqueous medium. This membrane was prepared by polymerization in a narrow mold and exhibited a thickness of 1 millimeter in the swollen state. Fig. 10B–D shows the AFM images of the surface structures, where the distance between the corners of neighboring rectangular gold nanoparticles was actively controlled by gradual swelling of the supporting pAAc membrane.

**Fig. 11:**
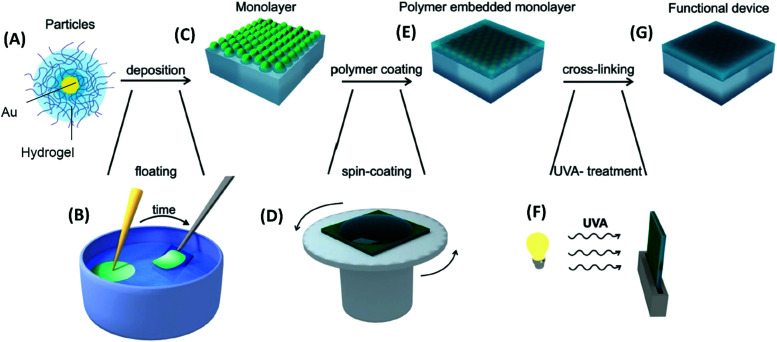
Flow diagram for (A) pNIPAAm-capped gold nanoparticles that are (B) self-assembled at the liquid–air interface and (C) transferred to a solid substrate followed by (D)–(F) embedding into a polymer layer. Adapted with permission from ref. 131.

In the second approach, the employed metallic nanoparticles are prepared by chemical synthesis. Research in this field lead to the development of a plethora of protocols enabling precise control of the particle morphology ranging from spherical, rod, core–shell to hyperbranched shapes.^134^ These metallic nanostructures can be either capped by a responsive hydrogel,^135^ embedded in small hydrogel particles (microgels or nanogels)^136^ or formed inside the already prepared responsive polymer network structure.^137^ These systems can be subsequently assembled as individual building blocks into the desired periodic^138^ or more complex^139^ geometries. A common technique takes advantage of the self-assembly process of such nanoparticles occurring at the liquid–liquid or liquid–air interface.^110^ Thereby, periodic two-dimensional hexagonal arrays are typically prepared. The schematics in Fig. 11 illustrate such an assembly process with subsequent transfer to a solid substrate and further processing of the monolayer (embedding in polymer layer and photocrosslinking) to yield the final optical device.^131^

**Fig. 12:**
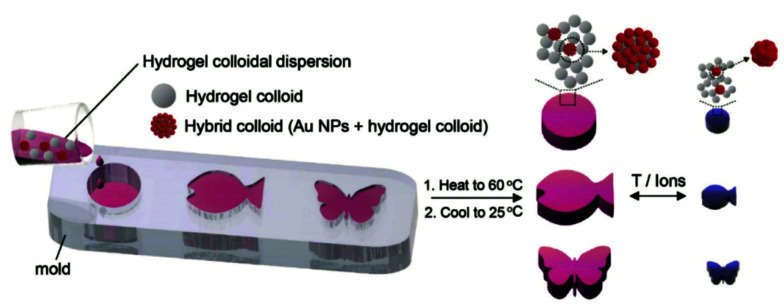
Preparation of plasmonic hydrogel objects with tunable optical absorption by casting responsive microgels with and without gold nanoparticle decoration in an appropriate mold. A color change due to plasmonic coupling of the gold nanoparticles is observed upon swelling and collapsing of the responsive polymer colloids upon an external temperature or ionic stimulus. Adapted under CC-BY from ref. 77.

In the third possible approach, the chemically synthesized metallic nanostructures are either attached to the object surface or dispersed within the polymer matrix upon the formation of the hydrogel. When molding the hydrogel by polymerization in cavities, the size of the resulting responsive devices is typically >10 μm and the metallic nanoparticles are trapped inside the crosslinked polymer network.^140^ Alternatively, preformed responsive microgels can be employed as building blocks that are decorated with metallic nanoparticles. From these raspberry-like structures, the bulk hydrogel material is formed by casting inside the mold, see the example in Fig. 12.^77^

Responsive hydrogel structures with sub-micrometer features were obtained from photocrosslinkable polymers on the top of plasmonic substrates by more precise lithography methods. For example, arrays of 100 nm-wide responsive hydrogel nanopillars were prepared by UV-NIL with nanostructured soft polymer stamp casted to a layer of photocrosslinkable polymer.^141^ Furthermore, by crosslinking a thin pNIPAAm-based polymer film with a UV interference pattern, responsive hydrogel relief gratings with a period <300 nm could be attached to a flat gold film.^142^ Similarly, this approach was adopted by using four-beam UV-LIL for the preparation of non-connected arrays of responsive pNIPAAm-based hydrogel domains with a diameter <200 nm that were wrapped around lithographically-made gold nanocylinders.^118^

Let us note that there is often the necessity to chemically link responsive polymer networks to the surface of the metallic or oxide nanostructures in order to yield a stable system. For the networks that are prepared *via* photocrosslinking of polymer chains in contact with these nanostructures, linker molecules bearing thiol headgroups can be employed that form self-assembled monolayers on most commonly used gold surfaces (see Fig. 6C) or silane headgroups reacting with oxide materials. For such photoactive groups, benzophenone^143^ or anthraquinone molecules^144^ can be used to simultaneously attach and crosslink the polymer chains to plasmonic or other type of optical structures upon irradiation with UV light^143,145^ or by exploiting a two-photon absorption process.^144^

### 3.3 Plasmonic heating

**Fig. 13:**
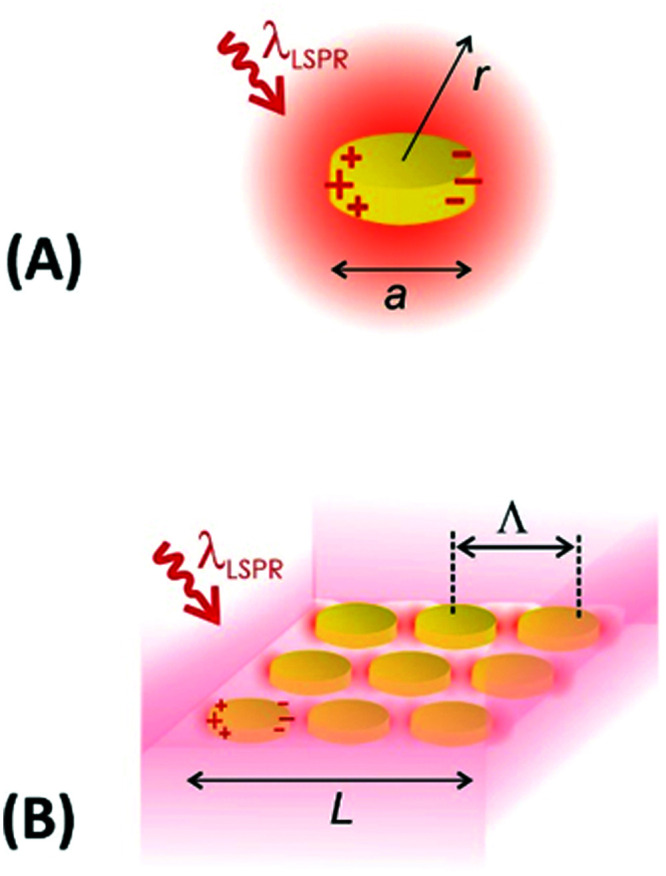
Schematics of plasmonic heating by energy dissipation of optically excited LSPs for (A) an individual nanoparticle and (B) collective heating of closely packed plasmonic nanoparticle arrays.

As numerous thermoresponsive polymers have been discovered, among possible stimuli available for actuating responsive hydrogels temperature is arguably the most commonly used. A temperature change stimulus Δ*T* can be applied with electronic devices such as Peltier elements or resistive microheaters. However, these devices typically allow for temperature control of macroscopic bulk volumes and thus are inevitably slow and not suitable for rapid actuating of miniaturized responsive hydrogel structures. An attractive solution to this problem offers a technique that is referred to as plasmonic heating.^146^ It allows for rapid and spatially confined heating of small volumes that comprise metallic nanostructures by using optical excitation of LSPs and their dissipation to heat by Ohmic losses. The rate of heating and dissipation of the heat to the surrounding medium depends on the particular design of the plasmonic structures. As illustrated in Fig. 13A, irradiation of an individual metallic nanoparticle leads to harvesting of the light energy followed by its conversion to heat with an absorption cross-section of *σ*_abs_. Then, the nanoparticle functions as a local heat source, and the temperature locally increases at its surface by:1
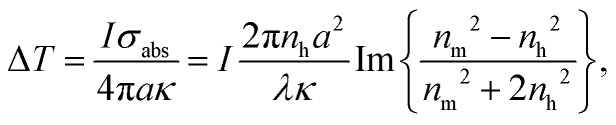
where *I* is the irradiation intensity of the heating light beam that impinges with a wavelength *λ* on a metallic nanoparticle with size *a* ≪ *λ*. The generated heat is dissipated to the surrounding dielectric medium with thermal conductivity *κ* and density *ρ*. The right-hand-side of the eqn (1) holds for the *σ*_abs_ of a spherical metallic nanoparticle with a diameter *a*, metal refractive index *n*_m_, and the refractive index *n*_h_ of the surrounding dielectric material. The temperature change Δ*T* decreases with the distance *r* from the nanoparticle as 1/*r* (for *r* ≫ *a*) and responds to changes in the irradiation intensity *I* with a time constant:2
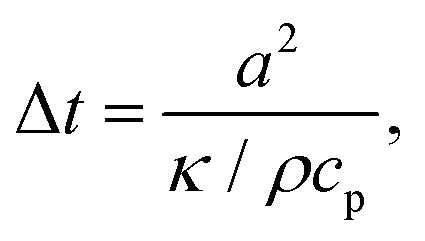
where *c*_p_ is the heat capacity. The factor *κ*/*ρc*_p_ is of ∼10^−3^ cm^−2^ s^−1^ for a structure surrounded by water that is kept at ambient room temperature.

Importantly, the time response Δ*t* and temperature change Δ*T* can differ substantially when ensembles of nanoparticles are used, as a collective heating effect may occur. Assuming 2D metallic nanoparticle arrays that are arranged with a period *Λ* and which are irradiated by a light beam with a footprint diameter of *L*, the contribution by collective heating become important. These two regimes can be distinguished *via* the following dimensionless parameter:3
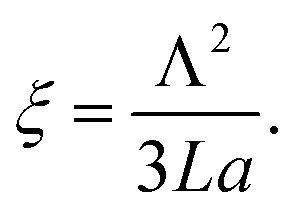
For large *ξ* > 1, each nanoparticle serves as an independent source that quickly heats only its close vicinity. However, the collective heating effect becomes dominant for small *ξ* < 1 and is accompanied by a stronger increase of temperature Δ*T* over the whole irradiated area with a footprint diameter *L*. Consequently, the time constant Δ*t* is then substantially prolonged to:4
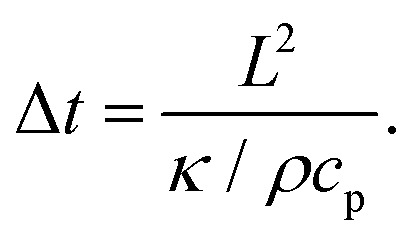


### 3.4 Plasmonic characterization of responsive hydrogel films and microgels

The metallic nanostructures embedded or contacted to responsive hydrogels allow for the optical measurement of the polymer network characteristics, including volumetric changes, the swelling ratio *Q*_V_ and the polymer volume fraction *Φ*_p_. These can be measured in equilibrium for static structures, but also their optical interrogation can provide a means for rapid probing of the time-dependent swelling and collapsing process of the polymer networks.

**Fig. 14:**
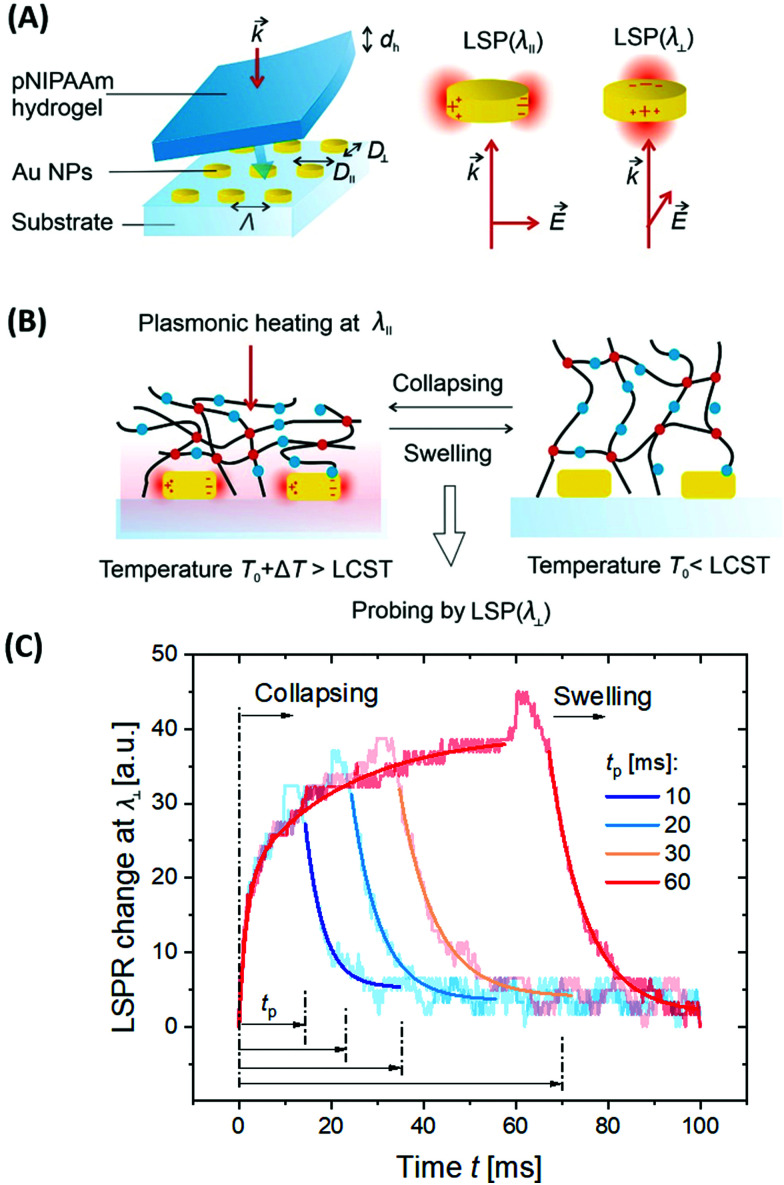
(A) Schematic illustration of the optical structure designed to monitor (B) rapid swelling and collapsing of NIPAAm-based polymer networks with (C) example of the obtained kinetics. Adapted with permission from ref. 148.

The detuning of the LSPR wavelength on arrays of metallic nanoparticles due to a refractive index change Δ*n*_h_ was exploited for rapid monitoring of swelling and collapsing of thermoresponsive polymers occurring within the tightly confined surface plasmon field. This approach offers the possibility to rapidly heat *via* LSP excitation and simultaneously probe the swelling state utilizing the same metallic nanostructures. In a recent work, arrays of elliptical-shaped nanoparticles were prepared by EBL and employed to characterize the responsive properties of pNIPAAm polymer chains that were grafted to these structures.^147^ The collective heating effect was dominant, and irradiating a footprint diameter of *L* = 3 μm with a near-infrared heating beam allowed to generate temperature changes with a time constant of Δ*t* ∼ 16 μs. The pNIPAAm chains forming a brush with a thickness of 30 nm responded to a quick temperature variation across the LCST with a transition time of 160 μs. A similar approach was reported for the observation of pNIPAAm network layers with a thickness of 800 nm (in the swollen state) that covered arrays of elliptical gold nanoparticles prepared by UV-LIL, see Fig. 14A and B.^148^ As seen in Fig. 14C, when monitoring the LSPR wavelength variations over time for the collapse (manifested as a gradual increase in LSPR wavelength) and swelling (seen as a gradual decrease of LSPR wavelength), transition times of several milliseconds were determined. The response of such pNIPAAm networks is substantially slower compared to the pNIPAAm brushes, which can be ascribed to the effect of the crosslink junctions in the polymer network, hindering diffusion of water molecules through the networks and giving rise to additional collective effects related to the imposed stress. Moreover, additional, substantially slower rearrangement of the polymer chains occurs simultaneously at longer time scales.^18^

Contrary to such slow transitions, a dramatically faster sub millisecond response was observed for gold nanoparticle aggregates with attached pNIPAAm chains.^149^ Such a fast transition was monitored for clusters of near field-coupled aggregated gold nanoparticles that expanded by the rapid (spring-loaded) conversion of pNIPAAm chains from a hydrophobic globular state to a hydrophilic stretched conformation. LSPR on metallic colloidal nanoparticles is associated with an enhanced scattering crosssection, which can be utilized to track their motion in suspension by dynamic light scattering (DLS). Furthermore, the influence of plasmonic heating on the diffusion behaviour of gold nanoparticles capped with a pNIPAAm hydrogel shell was studied by DLS.^92^ The observed effect was attributed to a change in local solvent viscosity as well as to a variation of the hydrodynamic radius by swelling and collapsing of the pNIPAAm shell.

LSPR supported by nanoscopic gold rods was also applied for the investigation of polymers responsive to other, different stimuli. Polyaniline (pANI) can be incorporated into hydrogels,^92^ which makes it responsive to pH changes and electro-oxidation/reduction. The electronic response was first investigated on arrays of cylindrical and rod-shaped gold nanoparticles prepared by EBL on an ITO substrate and coated with a 100 nm pANI film. A reversible wavelength shift of the LSPR by 70 nm was measured with a (non-optimized) switching time <10 s.^150^ To increase the magnitude of the LSPR wavelength shift, a particular architecture of chemically synthesized gold rod-shaped nanoparticles was proposed that were capped over their whole surface with a pANI shell of about 8 nm thickness. The structure enabled repeated shifting of the LSPR by 149 nm with the applied voltage.^151^ A similar approach to monitor the switching between a proton-doped and non-doped states upon changing the pH was reported for pANI-coated gold nanoparticles with a transition time of several tens of seconds.^152^

**Fig. 15:**
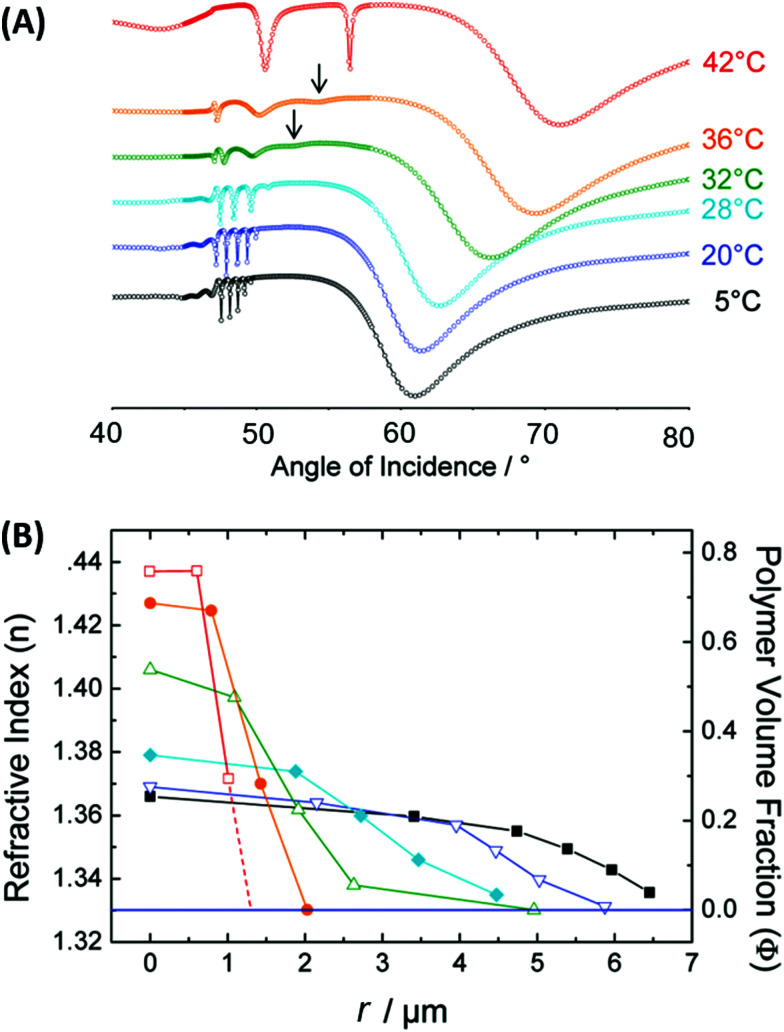
(A) Measured angular reflectivity spectra in combined SPR and OWS observation of the temperature-induced collapse of a pNIPAAm-based hydrogel film. (B) Reconstructed profile of the refractive index *n*_h_ and polymer volume fraction *Φ*_p_ as a function of a distance *r* from the interface, to which the hydrogel film is attached. Adapted with permission from ref. 55 ©2010, American Chemical Society.

Besides LSPR, also propagating surface plasmons – PSPs – can be utilized for the observation of responsive hydrogels optical systems with Kretschmann configuration that typically support SPR biosensors. For thin hydrogel films on flat gold surfaces, additional guided modes can travel along the polymer film as it exhibits a higher refractive index *n*_h_ than that of the adjacent aqueous medium. On this basis, SPR and optical waveguide spectroscopy (OWS) were combined to monitor the responsive characteristics of pNIPAAm-based hydrogels.^153^ The example in Fig. 15 shows angular reflectivity spectra for a pNIPAAm-based hydrogel film that gradually collapses upon increasing temperature *T* from below to above the phase transition of the polymer network. The density gradient of the hydrogel layer, represented by the polymer volume fraction *Φ*_p_, was reconstructed from the analysis of the resonances in the spectrum associated with the coupling of the incident light to PSPs and hydrogel optical waveguide modes (manifested as a series of narrow dips in the angular reflectivity in Fig. 15A). Moreover, OWS studies were employed to characterize pNIPAAm-nanoparticle composite films on solid supports,^98^ or free-standing membranes,^154^ that were tailored for blocking or opening the structure for the diffusion of target molecules to the plasmonic sensor surface. It is worth noting that swelling of surface-attached responsive hydrogel films is predominantly restricted to the direction perpendicular to the surface (1D swelling) due to lateral confinement by covalent bonding to the substrate. This effect establishes a strain along the interface that is partially released by buckling of the hydrogel surface, as observed from the increased scattering of light resonantly coupled to guided PSPs and hydrogel optical waveguide modes. Besides flat films, swelling and collapsing of periodic arrays of pNIPAAm features attached to a flat gold film were probed by PSP-enhanced diffraction,^141^ and by PSP Bragg scattering.^142^ These studies were performed in order to elucidate the different contributions by 1D swelling/collapsing in the direction perpendicular to the surface and by lateral swelling of the topographic features in the lateral direction, resulting in a quasi-3D swelling behavior.

## 4. Plasmonic (bio)sensors

### 4.1 Surface plasmon resonance-based readout

Plasmonic nanostructures, combined with responsive hydrogels, were employed in optical sensors and biosensors. The optical sensor response is generated by a physical stimulus or by specific interaction with the target species present in the analyzed liquid sample *via* induced changes in the responsive hydrogel characteristics. This type of optical readout can be implemented *via* functional moieties incorporated into the hydrogel polymer network in order to translate the specific interaction with a target analyte to variations in the crosslink density *v*_c_, to a change in the hydrophilicity of the network, or other effects that affect the swelling ratio *Q*_v_. In general, such stimulus-induced modulation of the swelling ratio *Q*_v_ can be converted to an optical signal *via* SPR probing of the associated refractive index changes δ*n*_h_ or through LSPR near-field coupling on the metallic nanostructures responding to hydrogel volumetric variations. These mechanisms were exploited in physical sensors (temperature, pH) as well as in chemical sensors and biosensors for the detection of molecular analytes related to medical diagnosis (glucose, cancer biomarkers).

**Fig. 16:**
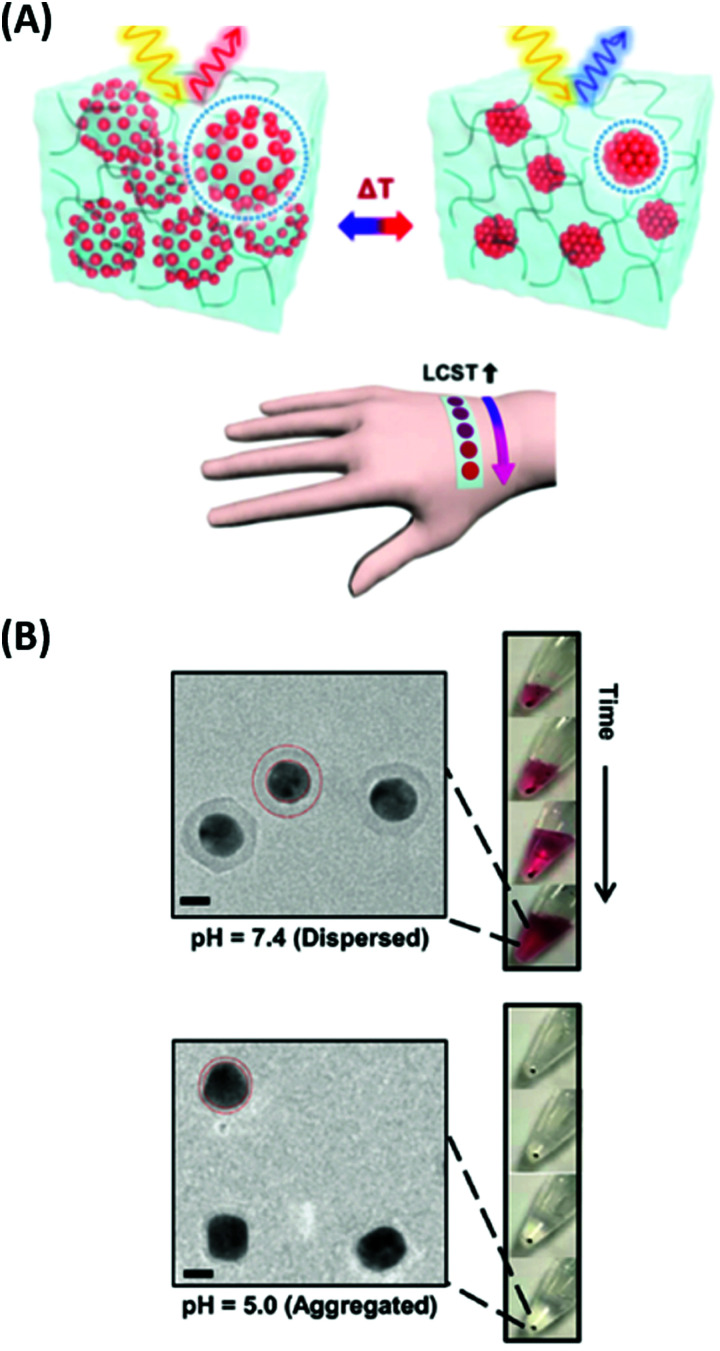
(A) Colorimetric sensor utilized by plasmonic microgels contacted with human skin and responding to variations in temperature. Adapted with permission from ref. 155. (B) TEM images of the deposited Au-CEA2 NPs in a dispersed and aggregated state that were used for the monitoring of intracellular pH. Scale bars: 10 nm. Adapted with permission of RSC from ref. 39.

pNIPAAm microgels conjugated with gold nanoparticles were implemented as plasmonic sensor elements in a smart wearable device that allows naked-eye readout of temperature variations in contact with skin (Fig. 16A).^155^ These stretchable sensor elements allowed to track temperature variations in the range between 25 to 40 °C within one second by the wavelength shift of the LSPR extinction peak due to the induced swelling ratio variation δ*Q*_v_ and a respective modulation of the distance-dependent LSP near-field coupling (see Fig. 16A). Another approach has been adopted to follow a pH change in the range from 1 to 12 by measuring the refractive index variations associated with swelling and collapsing of an acrylamide-based hydrogel.^156^ There, a fiber optic probe was used to observe the resonant excitation of PSPs in the transmission wavelength spectrum of a layered architecture comprising silver, indium tin oxide, aluminum, and a pH-responsive hydrogel. Monitoring of pH changes in very small volumes was shown recently.^39^ Here, gold nanoparticles capped with a thin layer of poly(methacrylic acid) (MAA) or poly(2-carboxyethyl acrylate) (CEA) were introduced to HeLa cells. Intracellular pH changes triggered the shell swelling (see Fig. 16B) and led to an aggregation of the nanoparticles accompanied by a red-shift and a broadening of the LSPR bands, observable as color changes in dark-field optical microscopy.

**Fig. 17:**
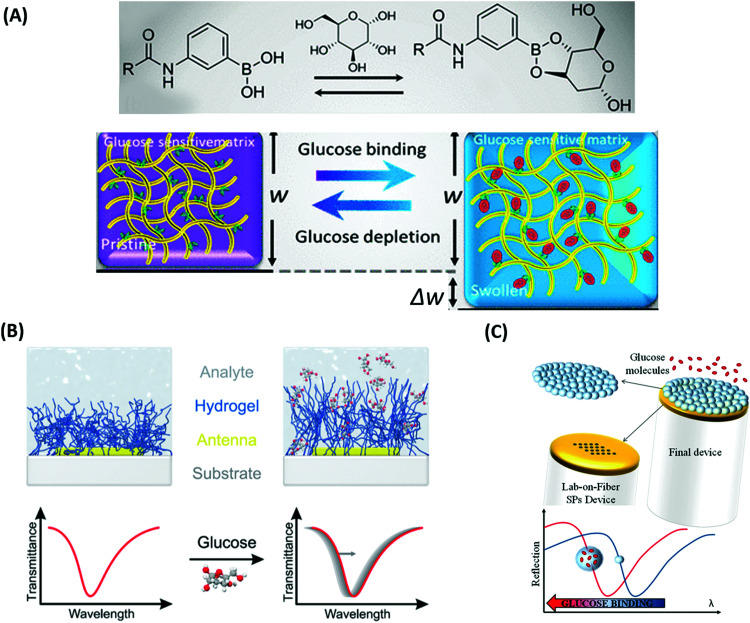
(A) Illustration of the glucose binding-induced swelling of the phenylboronic acid-functionalized hydrogel matrix that generates a change in the refractive index. Adapted under CC-BY, 4.0 from ref. 158. (B) Layout and sensing principle relying on plasmonic nanoparticles supporting LSPs that probe responsive hydrogel swelling due to a refractive index change induced by glucose binding. Adapted with permission from ref. 157, © 2015, American Chemical Society. (C) Detection scheme for probing with PSPs a glucose-responsive microgel matrix attached to a perforated gold film on an optical fiber tip. Adapted under CC-BY from ref. 159.

Sensors based on a hydrogel binding matrix with incorporated boronic acid moieties were reported for the selective detection of glucose molecules.^157^ The responsiveness derives from the reversible, pH-dependent formation of a boronate ester between the boronic acid and the *cis*-diol-motif present in glucose (see Fig. 17A),^58,158^ resulting in a transition of the hydrogel matrix from a lesser to a more hydrophilic state and consequently leading to a volumetric increase. This volume increase can be optically measured by the plasmonic response of gold nanoparticle arrays made by lithography and covered by a glucose-responsive HEMA-based hydrogel layer with incorporated boronic acid moieties. Upon binding of glucose molecules, the hydrogel swelled, and the LSPR wavelength was reversibly blue-shifted (see Fig. 17B). Additionally, the hydrogel matrix kept larger molecules away from the plasmonically probed surface, prohibiting unspecific binding of other constituents, like proteins, in medical samples and enabling the non-invasive continuous monitoring of this target analyte in tear fluid. The same interaction mechanism was employed in a glucose sensor, where a pNIPAAm-based microgel with 3-aminophenyl boronic acid moieties was deposited on a perforated metallic film at an optical fiber tip.^159^ Here, the binding of glucose was accompanied by a swelling of the gel and an increase in its *Q*_v_, which blue-shifts the wavelength at which PSP modes were resonantly excited at the fiber optic probe, as monitored in the reflected wavelength spectrum (see Fig. 17C).

**Fig. 18:**
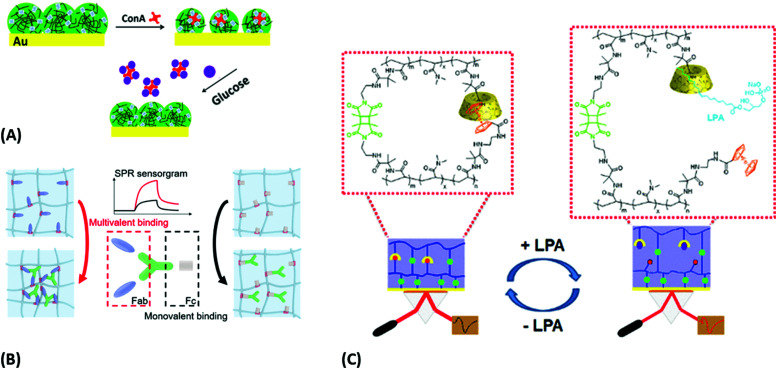
Plasmonic sensor concepts based on a modulation of crosslinking density: (A) platform with p(NIPAAm-*co*-glycosyloxyethyl methacrylate) microgels for glucose binding. Adapted with permission from ref. 160, © 2019, American Chemical Society. (B) Principle of multivalent protein binding and corresponding sensorgram, discriminating multivalent (left) and monovalent binding (right). Adapted with permission from ref. 161, ©2020, American Chemical Society. (C) Schematic of the sensor and its response mechanism based on disrupting β-CD and Fc complexes with target molecule LPA. Adapted with permission from ref. 163, ©2020, American Chemical Society.

Sensors relying on the variation of the crosslinking density *ν*_c_ mediated by target analyte-binding in responsive hydrogels were also utilized in another approach for the detection of glucose. In this modality, a displacement assay relying on the specific interaction of glucose with concanavalin A (ConA) moieties was exploited.^160^ For SPR readout, a poly(NIPAAm-*co*-glycosyloxyethyl methacrylate) microgel film was fabricated on a gold layer, which contracted upon addition of multivalent ConA due to an increase of the crosslinking density *ν*_c_. Competitive binding of glucose from the analyzed sample by the incorporated ConA units inside the hydrogel led to a disruption of the ConA crosslinks, subsequent microgel reswelling, and an increase in *Q*_v_ accompanied by a decrease in the refractive index *n*_h_ (Fig. 18A).

A system with the reversed modulation of crosslinking density *v*_c_ was pursued with bioresponsive p(NIPAAm-*co*-AAc) nanogels carrying multivalent protein binding (MPB) moieties for detection of PD-1 proteins (Fig. 18B).^161^ Small multivalent cations (*e.g.*, Ca^2+^, Fe^2+^, and Fe^3+^) can be distinguished by SPR over monovalent cations (*e.g.*, Na^+^ and K^+^) as they form a protein complex with the analyte. Multivalent binding of polyclonal antibodies was also explored by using a pNIPAAm microgel to detect progesterone by the optical etalon principle.^162^ Like in the previous work, an increase in the hydrogel cross-linking density *ν*_c_ due to the specific interaction with the analyte was monitored *via* the induced changes in the hydrogel refractive index *n*_h_.

Another report communicated bioresponsive supramolecular hydrogels that were probed by SPR and OWS techniques (Fig. 18C).^25,163^ A reversible sensor platform exhibiting two different crosslinks was demonstrated: a covalent photocrosslinker and a host-guest recognition pair from β-cyclodextrin (β-CD) and ferrocene (Fc). Increased swelling in the presence of the small targets adamantane amine (ADA) and the cancer marker lysophosphatidic acid (LPA) occurred due to breaking of the non-covalent bond between β-CD and Fc, which led to a decrease in the refractive index *n*_h_. Monitoring these changes allowed to determine the analyte concentration with a limit of detection of 2.43 × 10^−5^ M for ADA and 2 μM for LPA under plasma-like conditions. The achieved results document the potential of this approach to establish a new direction for small analyte sensing with direct detection format.

**Fig. 19:**
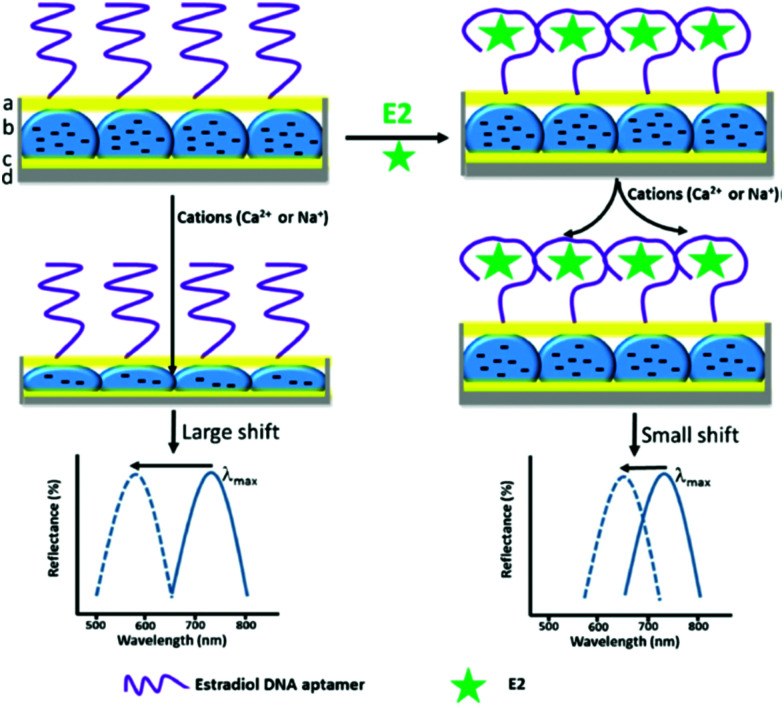
Schematics of an optical etalon showing the sensing mechanism, with the DNA aptamer binding estradiol 17β (E2) and forming a specific secondary structure that blocks Na^+^ or Ca^2+^ from entering the etalon microgel layer. The extent of blocking is directly proportional to the concentration of E2 in the sample, as documented by the typical reflectance spectrum obtained from this optical setup. Adapted by permission from Springer Nature, ref. 164, ©2018.

An alternative sensing principle was proposed with an ionoresponsive hydrogel for the detection of estradiol 17β in milk samples by a Fabry–Perot etalon-based sensor system.^164^ In this setup, a responsive microgel layer was sandwiched between two metallic layers, with the outer layer being porous and modified with a DNA aptamer. The affinity interaction of the aptamer with the target analyte estradiol 17β led to blocking of the pores and a hindered diffusion of Na^+^ or Ca^2+^ ions into the cavity with the responsive microgel. When estradiol 17β was bound to the aptamer, ion diffusion and subsequent collapse of the microgel layer was hindered, which could be observed as a change in the Fabry–Perot fringes. In this detection scheme, the external stimulus of the responsive hydrogel is spatially separated from the specific analyte recognition (see Fig. 19).

### 4.2 Plasmonically-enhanced optical spectroscopy readout

Besides probing the changes in refractive index *n*_h_ and *Q*_v_ that occur inside responsive hydrogels designed to specifically interact with the target analyte, these materials can also serve in optical spectroscopy-based detection of species trapped in their network. The responsive hydrogel material is typically utilized to enhance the collection efficiency of target analyte from the liquid sample capitalizing on the large surface area of permeable 3D mesh structure of the polymer network. After trapping the analyte, the responsive hydrogel binding matrix can be collapsed with an external stimulus, and by this allows compacting the captured analyte in the vicinity to metallic nanostructures. These zones represent the most sensitive region of the sensor and thus the initially weak optical spectroscopy signal is then amplified by the hydrogel collapse. At this stage, the optical readout is performed by the confined surface plasmon field that exhibits increased intensity and is associated with enhanced local density of optical states. In addition, the possible reconfiguration of plasmonic nanostructures using responsive hydrogels offers attractive means to actively form plasmonic hotspots (volume where the optical field is tightly confined) for probing the locally captured target analyte with advanced readout sensitivity and reproducibility.

In surface plasmon-enhanced spectroscopy (PEF, also referred to as surface-enhanced fluorescence SEF), the plasmonic nanostructures are tuned to confine the electromagnetic field intensity at wavelengths that are coincident with the absorption and emission bands of chosen emitters. These emitters are typically organic fluorophores, serving as labels when conjugated with the biomolecules to constitute an assay for the target analyte. The possibility to post-modify the permeable structure of responsive hydrogels with functional biomolecules as ligands was pursued to increase the binding capacity of the sensor surface and thus improve the probability of the specific analyte capture.^91^ It should be noted that depending on the density of ligand molecules in the hydrogel matrix, the target analyte can diffuse inside the gel only a certain distance from its outer interface in contact with the analyzed liquid sample, which is typically several hundreds of nanometres.^165^ However, such thick binding matrices cannot be efficiently probed with the confined field of surface plasmons that probe depth below several tens of nanometres for LSPs or about a hundred nanometres for PSPs. Therefore, there was investigated a sensing concept, where the efficient analyte capture in the swollen gel state is followed by triggering the gel collapse in order to drag the analyte to a smaller volume probed with the confined surface plasmon field.

**Fig. 20:**
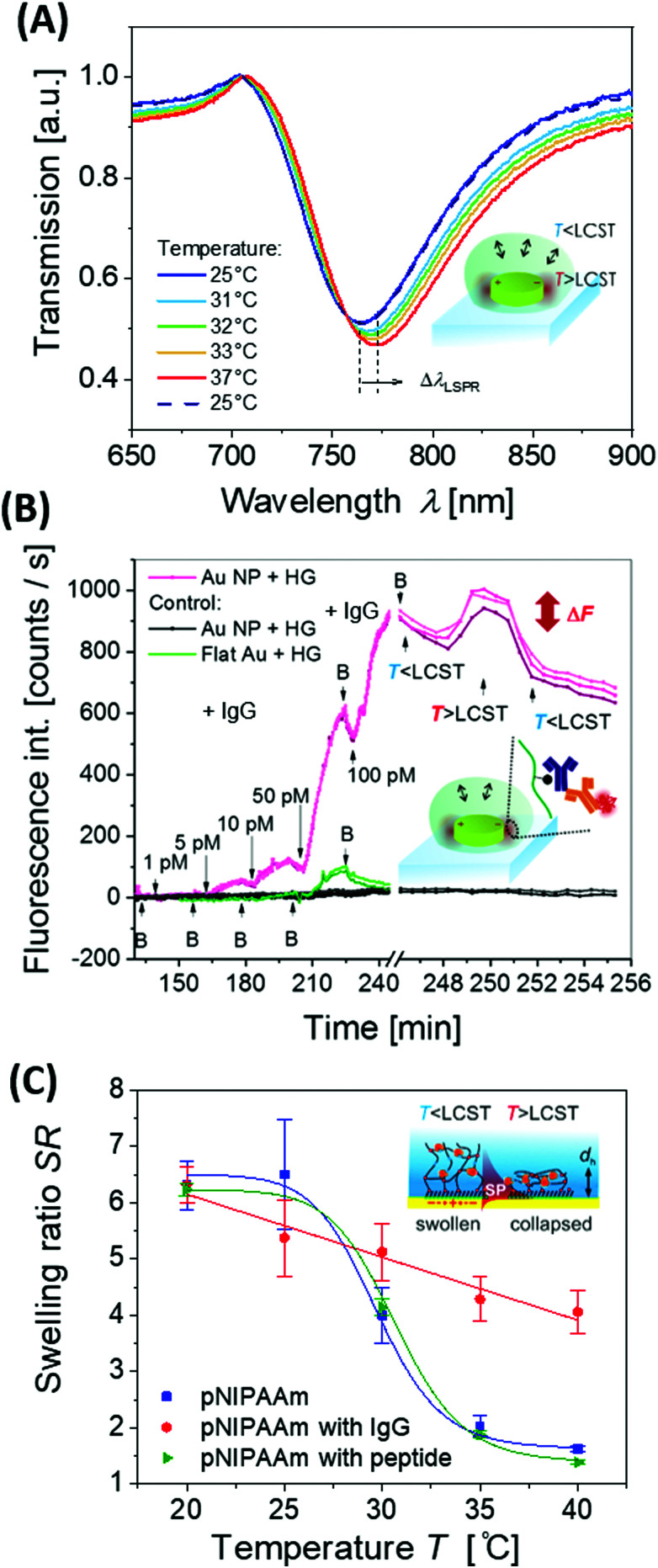
(A) Example LSPR spectra and (B) PEF readout upon affinity binding to an IgG-functionalized pNIPAAm binding matrix wrapped around gold nanoparticles in an arrays after active compacting of the captured analyte at the plasmonic hotspot by a temperature stimulus. Adapted under CC-BY 4.0 from ref. 118. (C) Changes in responsive properties of a pNIPAAm-based hydrogel layer due to its post-modification with a high molar mass protein ligand (IgG antibody) and a low molar mass peptide ligand. Adapted under BY-NC-ND 4.0 from ref. 167.

The realization of this sensing concept was reported for the immunoassay-based detection of IgG molecules labelled with Alexa Fluor 647 fluorophore using a pNIPAAm-based hydrogel binding matrix attached to a gold surface.^166^ The matrix exhibited a thickness of about 750 nm in the swollen state, and after the analyte capture it as collapsed to about 180 nm by increasing the ionic strength with 5 M NaCl solution. This collapse led to an increase in the plasmon-enhanced fluorescence intensity by a factor of about 3.1 when the interface was probed by PSP modes at the wavelength matching the fluorophore label absorption band. In order to utilize a more convenient temperature stimulus, an immunoassay detection scheme was developed that employs arrays of cylindrical gold nanoparticles wrapped with a pNIPAAm-based network of about 200 nm thickness in the swollen state, which compacts around the gold nanoparticles when exceeding the transition temperature of the polymer, Fig. 20A.^118^ The hydrogel network was postmodified with an IgG antibody ligand, and the affinity binding of target IgG antibody that was conjugated with a fluorophore was monitored by the LSP-enhanced fluorescence signal. As can be seen in Fig. 20B, the affinity binding manifests itself as a gradually elevated fluorescence intensity. After the affinity binding experiment, temperature *T* was increased above the phase transition temperature *T*_c_ of the unmodified pNIPAAm. However, a respective fluorescence signal intensity increase of only 20% was observed (marked as red arrow in Fig. 20B), which can be attributed to the fact that the thermoresponsive properties of the pNIPAAm-based network are severely impaired after the postmodification with large molar mass ligands such as IgG. By studying this effect in more detail, it was observed that the temperature-induced collapse of similar pNIPAAm layers led to a decrease in the swelling ratio *Q*_v_ by a factor of 4.7, but after its postmodification with IgG this ratio dropped to only 1.6.^167^ This work suggests that a possible route to circumvent this problem is using ligands with lower molar mass imposing smaller changes to the thermoresponsive characteristics of the pNIPAAm binding matrix. Fig. 20C shows for the polymer matrix modified with a smaller peptide ligand that the temperature-induced collapse led to a similar change in the swelling ratio *Q*_v_ by a factor of 4.3 as in the case of the pristine gel layer.

**Fig. 21:**
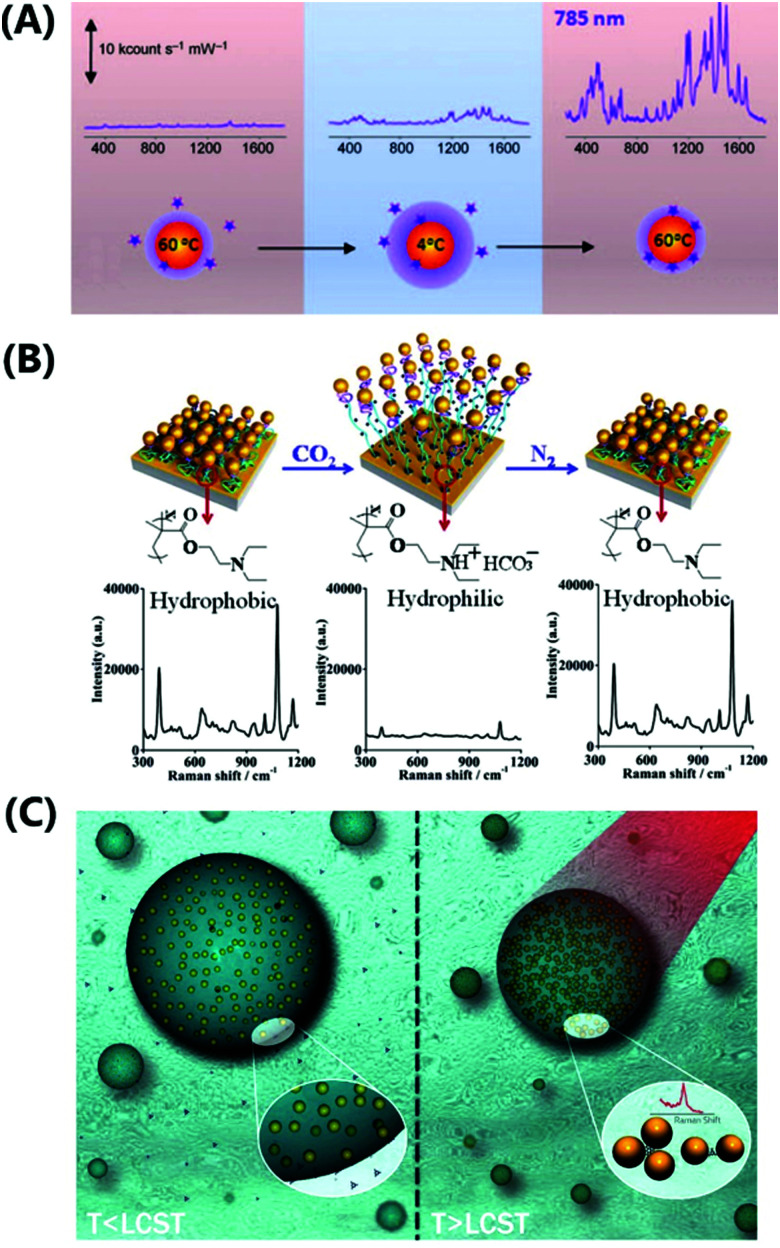
Overview of geometries utilized for the SERS detection that combine the responsive polymer and metallic nanostructure building blocks: (A) individual metallic nanoparticle capped with a pNIPAAm hydrogel shell for collecting and compacting of target molecules on its surface within the plasmonic hotspot. Adapted with permission from ref. 168. (B) Plasmonic nanoparticles attached to a flat metallic surface *via* a responsive polymer brush architecture that allows to control over their distance and formation of a tightly confined plasmonic hotspot. Adapted with permission from ref. 168 and 121. (C) Thermoresponsive microgel beads loaded with metallic nanoparticles for collection and trapping of a target analyte in the plasmonic hotspot formed upon the collapse of the network. Adapted with permission Royal Society of Chemistry from ref. 169.

At short distances *d* < 15 nm from the metal surface of plasmonic nanostructures, a strong effect of fluorescence quenching competes with the PEF. Therefore, small metallic nanoparticles that exhibit a tighter confined LSP field than PSPs on continuous metallic films are typically less efficient in PEF due to the strongly pronounced effect of quenching occurring at their close proximity. The effect of quenching of fluorescence was reported for colloidal silver nanoparticles with about 50 nm thick polymer shells based on a pNIPAAm hydrogel.^168^ In this study, the absorption wavelength of the selected emitters was tuned to match the LSP wavelength of the particles. It was shown that upon collapse of the pNIPAAm shell towards the surface of silver core nanoparticles, the fluorescence intensity of the hydrogel-associated emitters decreased in comparison to the swollen state. Nevertheless, this type of material is attractive for the amplification of weak Raman scattering signals and can serve as an efficient SERS substrate since it is not prone to quenching. Fig. 21A shows no measurable Raman signal when bringing Raman-active analyte molecules in contact with silver nanoparticles capped by a densely collapsed pNIPAAm shell at an elevated temperature. However, when decreasing the temperature below the phase transition, the hydrogel structure swells and becomes permeable for the Raman active molecule, which can diffuse to the silver surface, as observable by the appearance of Raman peaks. This signal is strongly enhanced by subsequent heating above the hydrogel phase transition, which drives the Raman-active molecules locked inside the polymer network towards the plasmonic hotspot at the silver surface. Interestingly, these data show that the pNIPAAm network itself does not show pronounced Raman peaks.

With the design of more complex architectures and engineering of the plasmonic hotspot, the Raman scattering efficiency by the plasmonic nanoparticles can be further increased. In order to achieve stronger confinement of the electromagnetic field (and respective enhancement of the field intensity), near-field coupling of metallic nanostructures has been investigated. The implementation of this concept was described in a recent publication with a layer of pNIPAAm microgels sandwiched between a flat gold layer and a permeable film of gold nanoislands.^170^ At temperatures below the hydrogel phase transition, the polymer network provides a permeable structure that allows the diffusion of target analyte molecules. Upon a temperature increase these molecules are trapped inside the hydrogel matrix by the microgel collapse, which also reduces the gap between the gold nanoislands and the flat gold film. In consequence, a plasmonic hotspot is formed that leads to an increased SERS signal. This concept was demonstrated for the detection of trinitrotoluene, l-tyrosine, and DNA at detection limits of 10^−11^ M. Similar phenomena were exploited by nanostructured gratings coated with a pNIPAAm layer for sensing of azobenzene dyes at a concentration as low as 10^−14^ M.^171^

Temperature-controlled near-field coupling between synthetically prepared gold nanoparticles and a flat gold film was reported with a pNIPAAM brush as a linker.^120^ The gold nanoparticles were decorated with a Raman-active methylene blue dye *via* click coupling. The SERS signal intensity of the system increased upon raising temperature above the polymer phase transition *T*_c_ through compaction of the pNIPAAm-based linker as the gap between the gold nanoparticles and the flat gold surface was reduced. A similar geometry was utilized to construct a CO_2_ sensor based on SERS readout.^121^ In this case, a diblock copolymer brush of poly(*N*,*N*-diethylaminoethyl methacrylate) (pDEAEMA) and pAAm was attached to the gold surface, followed by coupling of chemically synthesized gold nanoparticles. These nanoparticles were conjugated with 4-mecaptophenol to serve as Raman-active reporter, see Fig. 21B. When this sensor architecture was exposed to CO_2_ in aqueous medium, the pDEAEMA block was protonated and formed ion pairs with the carbonic acid, which led to an increased swelling. Consequently, the gold nanoparticles moved further away from the gold surface (from 15 to 26 nm) and a decrease in SERS signal was detected.

Another class of sensor architectures that allows modulation of the near-field coupling between metallic nanoparticles has been realized with microgels that were loaded with metallic nanostructures in their core, see Fig. 21C. Specifically, a pNIPAAm microgel with embedded 30 nm silver nanoparticles was employed as the SERS transducer.^172,173^ Temperature changes then modulate the swelling state of the thermoresponsive microgels and by this means the distance between the nanoparticles is controlled. Accordingly, there is increased the SERS signal originated from analyte molecules that infiltrated the microgel polymer network. An analogous increase of the SERS signal was observed with a scaffold of gold nanoparticles coated with pNIPAAm by trapping and locally concentrating analyte molecules in the plasmonic hotspots through a temperature increase.^169^ Limits of detection was determined for adenine (10^−6^ M), melamine (10^−5^ M), and 2-naphthalenethiol (10^−6^ M) in an aqueous solution. By employing gold nanorods with a copolymer coating of NIPAAm and *N*-vinyl-2-pyrrolidone, SERS enhancement upon a temperature-induced collapse was demonstrated with the herbicide diquat as analyte.^174^

In most of these examples, the SERS enhancement factor and the hotspot formation were tuned by a temperature variation and the associated collapse of the thermoresponsive microgels. An alternative approach was proposed by a combination of SERS and colorimetric readout for pH sensing operating in the range of 1 to 12.^175^ In this platform, the pH trigger acted on pANI-coated gold nanorods that were encapsulated in microgels of poly(ethylene glycol)diacrylate.

In order to increase the target analyte collection efficiency in the SERS-based detection, there was utilized its selective pre-concentration at the surface silver nanoparticles by their coupling to molecular-imprinted polymer cavities and thermoresponsive pNIPAAm.^176^ Binding and release of the target analyte rhodamine 6G was controlled by adjusting the pNIPAAm swelling state *via* the temperature. At 30 °C the target analyte was selectively captured inside the imprinted cavities with the highest affinity and the strongest Raman signal was observed, whereas at higher temperatures, this effect diminished. The sensor was able to detect the target analyte at concentrations as low as 10^−12^ M.

## 5. Thermoresponsive hydrogel actuators

Mechanical actuators have become ubiquitous tools in modern society, assisting where the precision or strength of the human hand is not sufficient or the environment does not allow human presence.^177^ They are commonly composed of rigid mechanical components that allow to perform a series of reproducible operations and thus provide specific functions in applications ranging from automatic door openers to robotic assembly of cars and precise surgery within the human body.^178^ Despite all those achievements, such hard robots possess inherent shortcomings,^179^ particularly in environments with continuously changing surroundings, where direct contact of the robotic structures with humans, animals and soft objects is envisioned. Such limitations are being addressed in the emerging field of soft robotics by employing, for example, functional soft materials that undergo shape changes upon an external stimulus. Hydrogels responsive to pH, ionic strength, electric field gradients, temperature and light have been investigated for this purpose. Light stands out among those stimuli since it can be remotely applied with high spatial-temporal resolution and fast switching times. Metallic nanoparticles that sustain LSPR and which are embedded within a thermoresponsive hydrogel composite allow the efficient absorption of light to induce rapid local plasmonic heating and an associated volume change of the thermoresponsive polymer network. The present chapter discusses how this volume change can be translated into a controlled motion in order to deliver mechanical work, followed by examples of selected applications of photothermally driven actuators.

### 5.1 Shape morphing

Responsive hydrogel objects can react to an applied stimulus by a homogeneous volumetric expansion and contraction or by more complex anisotropic transition with specific deformation of its shape. As discussed further below, the simple homogenous volume change of the object without altering its specific geometric shape can serve in applications such as opening and closing valves^99^ or for optical indicators.^46^ However, more complex transformations associated with controlled time-dependent shape morphing of responsive hydrogels are needed to accomplish more sophisticated functionalities such as artificial motility and other intricate mechanical motions.^180^

For sufficiently large objects composed of homogenous responsive hydrogel material, applying a spatially nonuniform stimulus can generate local deformations that lead to their shape morphing.^181^ Practically, a gradient of electrical^182^ or magnetic^183^ field intensity or spatially patterned illumination^76^ and local heating^153^ have been employed for this purpose. It should be noted that light is particularly well-suited as a spatially non-uniform stimulus due to the high resolution and fast switching characteristics of available optical systems. In general, the application of spatially nonuniform stimuli provides a convenient tool for operation of responsive hydrogel devices by simple manipulation methods. However, it can impose increasing complexity to the system needed to establish the locally confined trigger of the stimulus and often requires tracking of the specific location and positioning during deformation of the device for precise control.

**Fig. 22:**
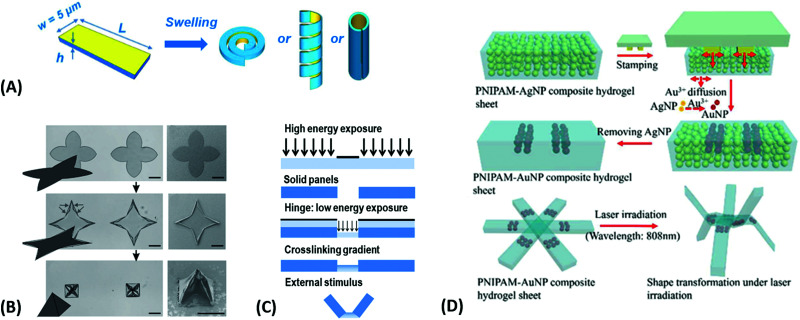
Shape morphing of devices composed of inhomogeneous responsive hydrogel material: A thermoresponsive hydrogel sheet is combined with (A) non-expandable gold layer (adapted under ACS AuthorChoice license from ref. 186 © 2017 American Chemical Society) and (B) a non-responsive hydrogel layer. (adapted with permission from ref. 184) (C) a system with locally varied hydrogel crosslinking density *ν*_c_, (adapted with permission from ref. 29 ©2014 IOP Publishing, Ltd), and (D) device with locally deposited absorbers enabling local heating of its thermoresponsive body. Adapted with permission from ref. 187 © 2013 Royal Society of Chemistry.

In another possible approach, a spatially uniform stimulus is employed to drive devices prepared from an inhomogeneous responsive hydrogel material. An architecture of this type studied in detail is the bilayer system composed of a rectangular thermoresponsive pNIPAAm sheet attached to a thin gold layer depicted in Fig. 22A. Depending on the ratio between length *L*, width *w*, and thickness *h*, swelling and collapsing of the sheet reversibly bends the structure with rigid metal layer along one of the main axes to adopt a spiral or cylindrical tube shape or, when bending along both main axes simultaneously, it can form a helical structure. Self-folding bilayer structures composed of stacks of pNIPAAm and pMMA hydrogel layers were designed to facilitate multiple folding steps.^184^ The resulting folding process is driven by the propagation of the diffusion front through the layers, which depends on the specific hydrogel material utilized. Tubular structures can be formed at the edges during the first folding step and act as reinforcements, while softer regions (for example, vertices) serve as hinges in succeeding deformation steps (see Fig. 22B). Photolithography allows controlled structuring of light-sensitive, photocrosslinkable polymers by spatially patterning the crosslinking density *ν*_c_. By combining stiff and highly crosslinked regions with less crosslinked softer sections that act as hinges, self-folding structures have been devised as illustrated in Fig. 22C and D. Self-folding hinge regions can be realized using a gradient in crosslinking density *ν*_c_ across the sheet thickness that causes a bending motion upon a temperature increase above the LCST of the employed thermoresponsive hydrogel material.^108^ A different approach for the self-folding of patterned pNIPAAm hydrogel devices was pursued using a nanocomposite material with spatially patterned plasmonic absorbers by locally embedding gold nanoparticles.^185^ To generate the pattern, a hydrogel stamp was soaked in HAuCl_4_ and contacted with a hydrogel with embedded silver nanoparticles. Gold ions diffuse into the composite and undergo a replacement reaction by locally converting silver nanoparticles to gold nanoparticles. Upon etching of the remaining silver nanoparticles using an aqueous solution of NH_3_ and H_2_O_2_, a patterned pNIPAAm-gold nanoparticle composite is obtained, see Fig. 22D.

**Fig. 23:**
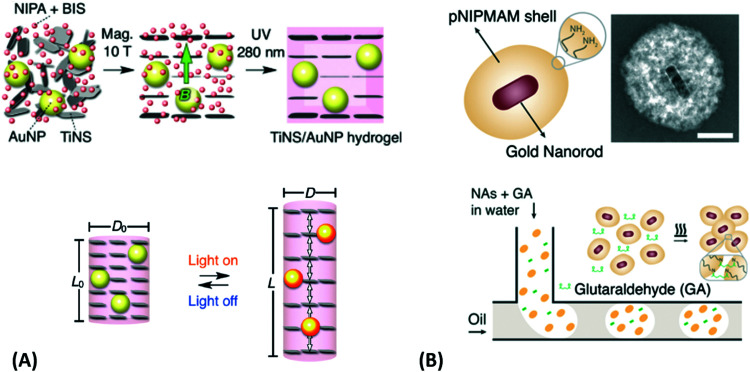
Shape morphing of composite materials relying on (A) thermoresponsive hydrogel system with anisotropic properties due to the embedding of oriented 2D nanostructures to a hydrogel polymer network. Adapted under CC-BY from ref. 33. (B) Nanoactuators formed by gold nanoparticles capped with responsive pNIPAAm-based shell are assembled to larger microactuators using microfluidics. Adapted under CC-BY-NC 3.0 from ref. 22.

Additional means to trigger shape morphing of responsive hydrogel devices are based on using anisotropic hydrogel composite materials that can be actuated by a spatially uniform stimulus. Anisotropic composites can be formed by embedding oriented 2D nanostructures inside the hydrogel matrix. Before gelation of the hydrogel body, these inclusions can be oriented by an electric field (graphene flakes)^188^ or magnetic field (titanate nanosheets)^33^ followed by their fixation in the gel. The embedded 2D nanostructures restrict expansion of the composite hydrogel material leading to anisotropic swelling with one preferred direction, as shown in Fig. 23A. Remarkably, the interaction between the nanostructure inclusions can significantly dominate the hydrogel swelling properties. For example, the nanocomposite of titanate nanosheets embedded in pNIPAAm hydrogel was shown to expand in one direction when increasing the temperature above its LCST – in stark contrast to regular pNIPAAm hydrogel materials that only shrink.^33^ The temperature-induced dehydration and rehydration of the hydrogel modulate the electrostatic permittivity, and thus, the repulsion between the inclusions leads to isovolumetric expansion. Since this mechanism is not associated with the diffusion of water molecules in and out of the polymer network, it can respond faster and is less limited by the overall size of the device.

An alternative route to accelerate the response time of thermoresponsive hydrogels, such as those based on pNIPAAm, employs a hierarchical architecture that minimizes the distance over which water needs to diffuse within the polymer network. This strategy was pursued for example in a bottom up approach where gold nanorods with a LSPR in the NIR part of spectrum were coated with a thermoresponsive poly(*N*-isopropylmethacrylamide) (pNIPMAM) shell that decreases in thickness when heated above the LCST at *T*_c_.^189^ The free amino groups in these building blocks were covalently crosslinked by glutaraldehyde inside a microfluidic device (see Fig. 23B). The obtained internally structured materials were employed as microactuators with dimensions between 5 μm and 50 μm, and were demonstrated to respond by two orders of magnitude faster than conventional homogenous bulk hydrogel devices of the same size.

### 5.2 Hydrogel valves

**Fig. 24:**
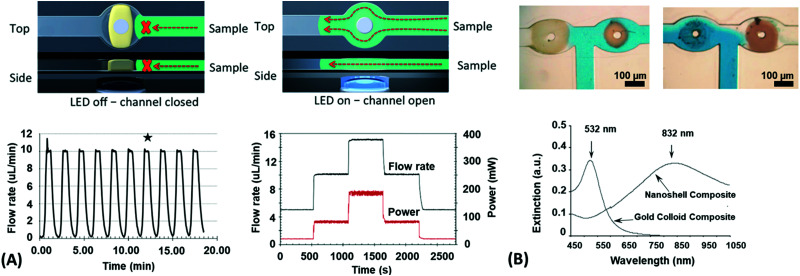
(A) Precise flow control through a microfluidic channel actuated by a photoresponsive hydrogel valve by modulating the irradiation power. Adapted with permission from ref. 190 ©2001 Royal Society of Chemistry. (B) Microfluidic T-junction with two photothermal responsive valves with embedded plasmonic nanoparticles with distinct LSPR wavelengths that can be independently actuated by using wavelength division multiplexing. Adapted with permission from ref. 109, ©2005 Wiley-VCH.

Responsive hydrogels found application in microfluidics for rapid and reversibly actuated valves needed in designs where conventional mechanical or hydraulic solutions are too bulky. Valves based on responsive gels can be designed such that fluid can flow freely through a microfluidic channel passed the collapsed gel, and upon application of the external stimulus, the expanding gel fills the channel and blocks the liquid flow. This concept was implemented by a thermoresponsive pNIPAAm-based hydrogel that was modified with spiropyran moieties to quickly respond to irradiation with light.^76^ The hydrogel valve schematically shown in Fig. 24A was deployed in a circular chamber with a 2.6 mm diameter that was connected to an inlet and outlet microfluidic channels with a width of 1 mm. This device was placed on top of a blue light-emitting diode, and by modulating the irradiation power, the swelling degree of the gel valves was tuned to regulate the flow rate through the valve. With a flow sensor and controller, a highly reproducible and stable flow control^72,190^ with a valve response time in the order of 10 s for increasing and 30–40 s for reducing the flow was demonstrated.

In general, optical actuation of such thermoresponsive hydrogels is beneficial for rapid and spatially addressable switching. Furthermore, it can be implemented into composite materials with dispersed metallic nanoparticles, capitalizing on plasmonic heating. They exhibit a higher absorption cross-section and, contrary to organic dye molecules, they do not photobleach. Selective actuation of microfluidic valves composed of such composite thermoresponsive material was demonstrated by wavelength division multiplexing.^109^ Two spatially separated valves were prepared from a thermoresponsive copolymer hydrogel based on NIPAAm and acrylamide, which comprised different plasmonic nanoparticles exhibiting spectrally distinct LSPR at a wavelength of 530 nm and 830 nm, respectively (Fig. 24B). Irradiating the microfluidic T-junction at these respective wavelengths allows to selectively open and close the integrated valves individually and thus direct the flow of liquid samples to the desired arms of the microfluidic channels. The response time of this system was below 5 seconds when illuminated with an intensity of 1.6 W cm^−2^ for 532 nm and 2.7 W cm^−2^ for 832 nm.

### 5.3 Hydrogel grippers

The ability to grasp and move objects is a crucial task of robotic applications at all length scales. Conventional robotics uses stiff mechanical grippers, suction cups, or magnetic grippers to pick up objects. The actuators are typically designed to manipulate specific types of objects and struggle with handling objects of arbitrary shape. Soft robotics addresses these challenges by employing actuators that are sufficiently flexible to adapt with respect to the object shape and they ensure a firm grip by switching between an elastic and a stiff state. On a macroscopic scale, hydraulic actuators offer fast response times with high force levels while maintaining compatibility with standard hydraulic or pneumatic controls. While the response time of stimulus-responsive hydrogels is severely limited in macroscopic devices (size exceeding millimeter scales), they can still provide unique opportunities when using hydraulic actuation. It was recently demonstrated that such actuators made of polyacrylamide (pAAm) can provide an advantage in camouflaged optical or sonar observations,^191^ capitalizing on the fact that the refractive index and the sonic impedance of such gels closely match the properties of the surrounding medium. Such a hydraulic actuation mechanism requires physical connecting of the device to a driving unit and consequently the implementation of stimuli-responsive hydrogels that can be controlled remotely have been found more attractive.

**Fig. 25:**
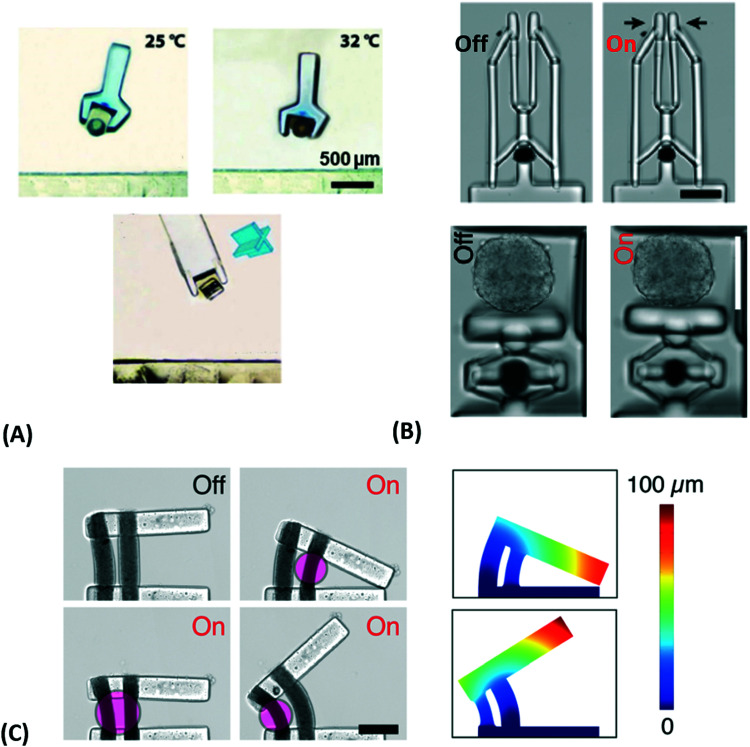
(A) A universal microgripper based on a hierarchal body is composed of crosslinked pNIPAAm microgel that allows pickup of complex shaped objects and is integrated into a magnetically steerable microbot. Adapted with permission from ref. 26. ©2018 Wiley-VCH. (B) Microactuators that drive a parallel jaw mechanism (top) and a pressure plate (bottom). Adapted under CC-BY-NC 3.0 from ref. 22. (C) A mechanical device composed of two extruded microactuator pillars, which is capable of a range of motion motifs (scale bar of 100 μm). Adapted under CC-BY-NC 3.0 from ref. 22.

For example, an universal microgripper based on a dual-network microgel composed of the shape-memory polymer sodium alginate and the thermoresponsive hydrogel pNIPAAm as been presented.^192^ The microgel is brought in contact with the object conforming to its shape. The shape is then locked by the shape-memory component of the gel by addition of Ca^2+^ ions that crosslink guluronic acid blocks in the sodium alginate by ionic bonds. To further tighten the grip, the temperature is then increased above the LCST of the thermoresponsive pNIPAAm component of the microgel, compressing the mixed polymer network. The process is fully reversible by a decrease of temperature and removal of the Ca^2+^ ions by either addition of ethylenediaminetetraacetic acid (EDTA) that coordinates with the Ca^2+^ or the introductions of ion such as Na^+^ and K^+^ that replace the Ca^2+^. Microgel spheres of about 500 μm diameter have shown holding forces up to 400 μN on a spherical object, sufficient to carry metallic objects of this size. The shape was fixed within seconds upon introduction of Ca^2+^ and the original shape of the gripper was restored within 2 minutes after introduction of EDTA, fully releasing the gripped object. Attached to a lithographically prepared magnetically movable body as shown in Fig. 25A, and integration of plasmonic absorbers into the hydrogel for remote heating, the fully remotely controlled gripper was used to repeatedly grab and move objects without signs of degradation.

A modular approach to the design of smaller and more complex mechanical devices with sub-millimeter dimensions and photothermal actuation was explored by using maskless projection photolithography.^189^ The active part of the systems was made of a composite hierarchical material with crosslinked nanoactuators (see Fig. 23A) that can be rapidly contracted by irradiation with light at a wavelength resonant with the embedded plasmonic nanoparticles. This material is mechanically coupled with other elements prepared from a non-responsive material based on poly(ethylene glycol)diacrylate. The examples in Fig. 25B show a parallel jaw gripper and a microscale piston, compressing a spheroid of epithelial cells. These systems were demonstrated to be remarkably durable, with the piston performance unchanged after 1000 cycles. Using an additive manufacturing approach, the crosslinked nanoactuators were encapsulated in an ionically-crosslinked porous alginate gel and extruded to form pillar-shaped ‘muscles’. Combining two such artificial muscles with a nonresponsive bar, shown in Fig. 25C, allowed to perform a range of motions. Plasmonic heating of a single actuator pillar leads to tilting, while simultaneous illumination of both actuator pillars induces movement of the lever parallel to the base. Remarkable are the observed response times of actuator pillars with 50 μm diameter that exhibit 80% contraction within 25 ms and subsequent expansion to 70% of original size within 0.5 s.

### 5.4 Hydrogel swimmers

**Fig. 26:**
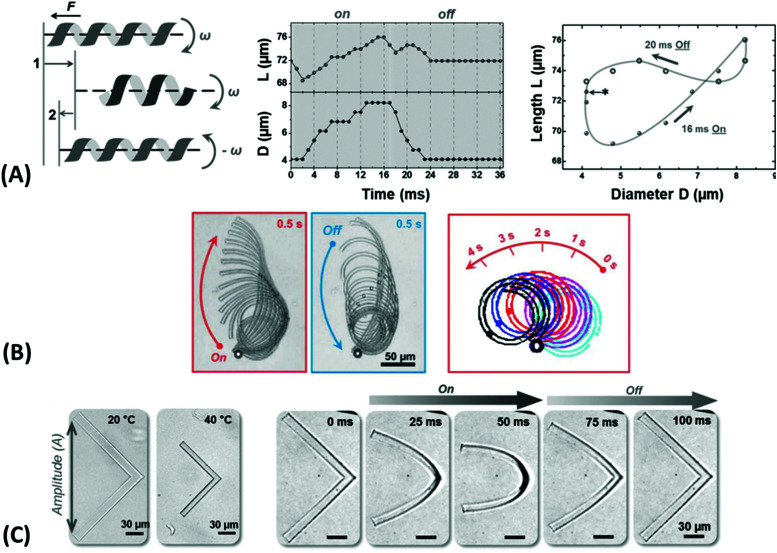
(A) Forward movement of different hydrogel ribbons in response to photothermal heating. Adapted with under CC-BY 4.0 from ref. 140. (B) Expansion and shrinking of a helical hydrogel ribbon resulting in rotation and net forward movement. Adapted under CC-BY from ref. 24 (C) L-shaped hydrogel structure showing (left) isotropic change in dimensions for slow temperature modulation and (right) forward movement resulting from fast photothermal heating. Adapted with under CC-BY 4.0 from ref. 140.

Propelled microbots attract particular attention due to their potential applications in the biomedical field.^193,194^ At micrometer length scales, such devices have to operate in aqueous environments in the regime of low Reynolds number, where the viscous resistance dominates and repeated shape morphing allows for propulsion only if non-reciprocal forward and backward transformation occurs. In this context, thermoresponsive hydrogels with embedded plasmonic absorbers were investigated as actuators. With such materials, a microswimmer based on a helical bilayer system (see Fig. 22) was devised based on torsional transformation upon swelling and deswelling.^140^ By rapid heating of the device at a time scale that is faster than the water diffusion-driven swelling and shrinking of the pNIPAAm hydrogel body, non-equilibrium transitions accompanied by a nonreciprocal transformation are induced, resulting in rotation along the two main device axes and performing a net forward motion in a confined channel, see Fig. 26A. A similar autonomous motion was observed for a semi-helical device that can perform rotations (see Fig. 26B).

In another design approach for devices capable of translational motion, shape morphing by rapid heating of an L-shaped ribbon made of thermoresponsive hydrogel with an arm length of 100 μm, a width of 10 μm, and a thickness of 5 μm (see Fig. 26B) is exploited.^140^ During slow temperature modulations that allow equilibrium swelling and shrinking, the hydrogel device does not alter its geometrical shape, but only its overall dimensions. However, when rapid plasmonic heating and cooling is implemented by a series of laser pulses with a wavelength of 808 nm and an intensity of 1.7 W mm^−2^, a time-dependent-deformation is induced by the non-equilibrium kinetics of water diffusion into and out of the polymer networks, associated with stress in the hydrogel material. This is demonstrated in Fig. 26C, where the L-shaped device legs are bent faster during the contracting phase of the heating cycle with the optical beam on than the relaxing phase upon cooling when the irradiating beam is switched off. These anisotropic deformations result in nonreciprocal motion and respective progressive movement.

### 5.5 Hydrogel crawlers

Another format of motile hydrogel microsystems is realized with worm-like robots, which are designed to move by mimicking peristaltic motion by applying a time-dependent, spatially nonuniform stimulus. This mode of actuation can be achieved *via* structured illumination of a homogenous responsive hydrogel system to reduce friction upon local contraction and allowing part of the body to move while the remaining body stays at rest. Propagating the deformation along the body in cycles provides progressive forward movement.

**Fig. 27:**
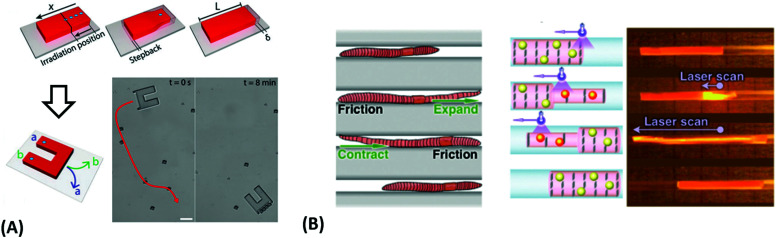
(A) Light-driven crawler actuated by peristaltic deformation of a pNIPAAm hydrogel with embedded gold nanoparticles upon non-uniform irradiation, which induces modulation of friction between the polymer network and the solid substrate. Scalebar = 100 μm. Adapted with permission from ref. 195, ©2019 Mary Ann Liebert Inc. (B) Microbot mimicking worm-like motion based on an anisotropic hydrogel composite that locally responds to nonuniform irradiation. Reproduced with permission from ref. 33, ©2018 Wiley-VCH.

For this purpose, the adhesion of pNIPAAm-based hydrogel devices on a glass surface was studied.^195^ In sliding experiments on a tilted surface under heating cycles, the friction of the contracted state was found to be significantly reduced compared to the swollen state. A worm-like microbot was then devised consisting of pNIPAAm hydrogel composites with embedded gold nanoparticle absorbers for photothermal actuation. Locally confined irradiation of only a part of the device with a focused laser beam induces local plasmonic heating and shrinking of the irradiated part, as shown in Fig. 27A. By scanning the laser beam over the body (100 μm × 50 μm × 30 μm), the region of reduced friction upon shrinking propagates along the main axis of the device yields net forward motion. Repeated unidirectional scanning of the laser beam transforms the device into a light-driven linear motor. Two such motors were combined to form a remotely controlled, steerable microbot used to collect cargo on a surface.

A relatively fast-moving microbot with dimensions in the millimeter range was demonstrated with an earthworm-like crawler^33^ built from a nanocomposite of cofacially aligned titanate nanosheets and plasmonic gold nanoparticles embedded in a pNIPAAm hydrogel. The charged nanosheets constrain swelling of the gel in two dimensions and add strain by electrostatic repulsion. By increasing the temperature, electrostatic permittivity variations of the hydrogel close to its LCST at *T*_c_ are induced that modulate the electrostatic repulsion between the nanosheets and subsequently lead to a uniaxial expansion of the structure normal to the nanosheet planes (see Fig. 23B). Contrary to regular pNIPAAm-based devices, this deformation occurs without expelling water from the device, so due to volume conservation, the uniaxial expansion is accompanied by the contraction along the two orthogonal directions. Scanning a laser beam along the device body translates into a peristaltic motion (Fig. 27B) through sequential, local expansion along the scan direction with contraction in the perpendicular directions. As a result, the illuminated segment moves forward against the friction of the non-irradiated segments of the device. The large expansion of up to 70% perpendicular to the nanosheets allows a 15 mm long hydrogel cylinder with 1.2 mm diameter to move by 7 mm, almost half the body length, within a single scan at a speed of 3.4 mm s^−1^.

## 6. Summary, conclusions and outlook

In this review, we take a deeper look into the properties of responsive hydrogels and plasmonic systems by firstly discussing the requirements, architectural adjustments, and their broad range of applications. We then focus on the combination of the intriguing characteristics from both material classes to yield plasmonic nanomaterials with responsive polymer hydrogels and the exploitation of their exceptional synergetic properties in applications, such as sensing and actuation.

**Fig. 28:**
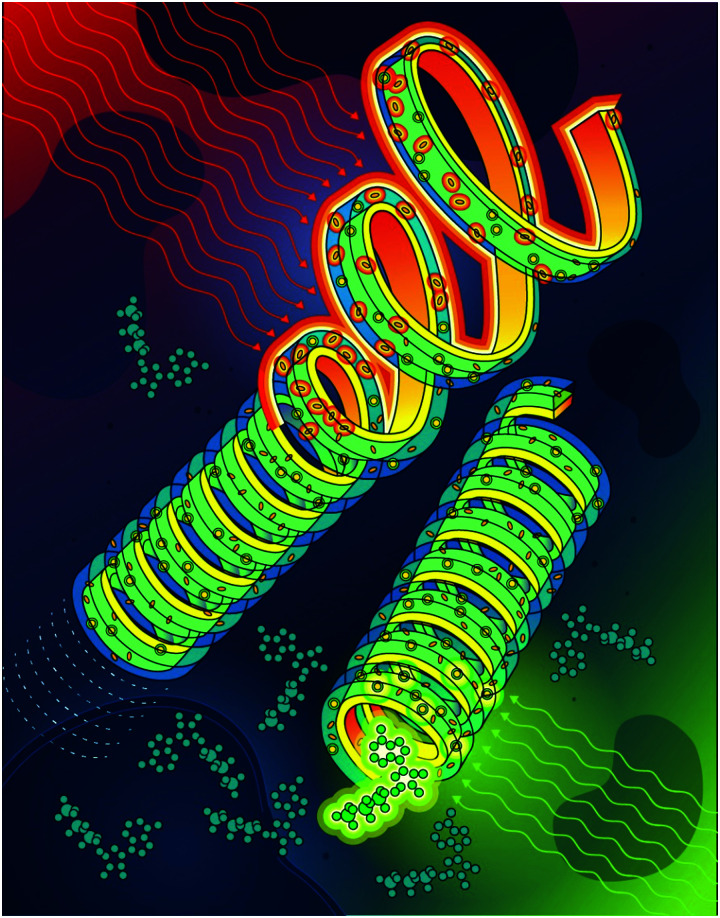
Schematics of a hypothetical microdevice that performs locomotion and is equipped with a biosensing functionality to detect the spatial and temporal concentration change of monitored target species.

In the light of the intriguing examples presented in this review, we witness a substantial increase in complexity and functionality of advanced soft materials, which are continously developed and exploited in a wide range of technical, biological and medical applications. Among these, responsive hydrogels stand out as they can transduce minute physical and chemical changes in their environment, such as pH, ionic strength, temperature, illumination, or presence of specific target molecules, to an abrupt transition in their structure. Particularly in connection with metallic nanomaterials, responsive hydrogels provide highly adaptable optical properties forming an important class of ‘smart’ materials. They can be tailored for applications in modern types of analytical devices paving even the way to the analysis in close contact with the human body. Moreover, responsive hydrogels were employed in light-driven actuators that, up to now, mainly served in microfluidic devices. The majority of recent experiments have been aimed at demonstrating their key functionalities, including remote control of liquid flow, externally-driven locomotion, and manipulation of miniature objects present in aqueous samples. These advances were enabled by a range of fabrication technologies, including lithography, nanoimprinting, colloidal assembly, or rapidly developing additive manufacturing. Research has already established light-propelled microfluidic valves, mechanical grippers, crawlers, and swimmers and is now on the verge of developing functional soft microrobotic devices. The key motivation in these efforts is guided by future applications in the medical field, where microscopic devices prepared from biocompatible materials with length scales of small blood vessels may navigate through organs and perform delicate tasks autonomously or remotely. They may also serve as micro-factories, building structures of micrometer dimensions with unpreceded precision and complexity. Therefore, one can expect that efforts of merging the potential of light-driven remote actuation and sensing within the functionalities of an individual miniature soft device will gaining substantial momentum in the future (see Fig. 28). Such activities will provide new tools for research as well as for applications in important fields of medical diagnostics, drug delivery, or theragnostic.

## Abbreviations

ADA: Adamantane amine
CEA: 2-Carboxyethyl acrylate
ConA: Concanavalin A
EBL: Electron beam lithography
Fc: Ferrocene
FRET: Fluorescence resonance energy transfer
GEMA: Glycosyloxyethyl methacrylate
HEMA: 2-Hydroxyethyl methacrylate
LCST: Lower critical solution temperature
LPA: Lysophosphatidic acid
*l*_s_
: Segment length
LSP: Localized surface plasmon
LSPR: Localized surface plasmon resonance
MAA: Methacrylic acid
MPB: Multivalent protein binding
*n*_h_
: Refractive index (hydrogel)
NIPAAm: *N*-Isopropyl acrylamide
NP: Nanoparticle
OWS: Optical waveguide spectroscopy
pAA: Poly(allylamine)
pAAc: Poly(acrylic acid)
pAAm: Poly(acrylamide)
pANI: Poly(aniline)
pDEAEMA: Poly(2-(diethylamino)ethyl methacrylate)
PEF: Plasmon-enhanced fluorescence
pNIPAAm: Poly(*N*-isopropyl acrylamide)
pNIPMAM: Poly(*N*-isopropylmethacrylamide)
PSP: Propagating surface plasmon
*Q*_V_
: Volume swelling ratio
SEF: Surface-enhanced fluorescence
SERS: Surface-enhanced Raman scattering
SPR: Surface plasmon resonance
*T*_c_
: Cloud point temperature
TEM: Transmission electron microscopy
UCST: Upper critical solution temperature
UV-LIL: UV laser interference lithography
β-CD: β-Cyclodextrin
*Φ*_p_
: Polymer volume fraction
*ν*_c_
: Crosslink density

## Conflicts of interest

There are no conflicts to declare.

## Supplementary Material
